# An HLA-E-targeted TCR bispecific molecule redirects T cell immunity against Mycobacterium tuberculosis

**DOI:** 10.1073/pnas.2318003121

**Published:** 2024-05-01

**Authors:** Rachel L. Paterson, Marco P. La Manna, Victoria Arena De Souza, Andrew Walker, Dawn Gibbs-Howe, Rakesh Kulkarni, Joannah R. Fergusson, Nitha Charles Mulakkal, Mauro Monteiro, Wilawan Bunjobpol, Marcin Dembek, Magdalena Martin-Urdiroz, Tressan Grant, Claire Barber, Diana J. Garay-Baquero, Liku Bekele Tezera, David Lowne, Camille Britton-Rivet, Robert Pengelly, Natalia Chepisiuk, Praveen K. Singh, Amanda P. Woon, Alex S. Powlesland, Michelle L. McCully, Nadia Caccamo, Mariolina Salio, Giusto Davide Badami, Lucy Dorrell, Andrew Knox, Ross Robinson, Paul Elkington, Francesco Dieli, Marco Lepore, Sarah Leonard, Luis F. Godinho

**Affiliations:** ^a^Immunocore Ltd., Abingdon, Oxfordshire OX14 4RY, United Kingdom; ^b^Department of Biomedicine, Neurosciences and Advanced Diagnostic, University of Palermo, Palermo 90127, Italy; ^c^Central Laboratory of Advanced Diagnosis and Biomedical Research, Azienda Ospedaliera Universitaria Policlinico Paolo Giaccone, University of Palermo, Palermo 90127, Italy; ^d^National Institute for Health and Care Research, Biomedical Research Centre and Institute for Life Sciences, Faculty of Medicine, University of Southampton, Southampton SO16 6YD, United Kingdom

**Keywords:** tuberculosis, immunotherapy, HLA-E, T cell receptor

## Abstract

Peptides presented by HLA-E, a molecule with very limited polymorphism, represent attractive targets for T cell receptor (TCR)-based immunotherapies to circumvent the limitations imposed by the high polymorphism of classical HLA genes in the human population. Here, we describe a TCR-based bispecific molecule that potently and selectively binds HLA-E in complex with a peptide encoded by the *inhA* gene of *Mycobacterium tuberculosis* (Mtb), the causative agent of tuberculosis in humans. We reveal the biophysical and structural bases underpinning the potency and specificity of this molecule and demonstrate its ability to redirect polyclonal T cells to target HLA-E-expressing cells transduced with mycobacterial *inhA* as well as primary cells infected with virulent Mtb. Additionally, we demonstrate elimination of Mtb-infected cells and reduction of intracellular Mtb growth. Our study suggests an approach to enhance host T cell immunity against Mtb and provides proof of principle for an innovative TCR-based therapeutic strategy overcoming HLA polymorphism and therefore applicable to a broader patient population.

## Significance

Tuberculosis (TB) is a disease with a high global burden and for which there is a high unmet need given the limited advances in antibiotic or vaccine development in the last 100 y. Here, we generated a high affinity soluble T cell receptor (TCR) specific for a *Mycobacterium tuberculosis* (Mtb) peptide presented by the human leukocyte antigen (HLA) molecule HLA-E. Therapeutic targeting HLA-E peptide complexes overcomes HLA-restriction and is therefore applicable to a broad patient population due to HLA-E limited polymorphism. We show that TCR-bispecific molecules, comprised of an affinity-enhanced TCR fused to an anti-CD3-activating domain, specifically induce T cell–mediated killing of Mtb-infected cells. We therefore propose that donor-unrestricted TCR-based immunotherapeutic could be an effective way to target TB infections.

Each year approximately 10 million people develop tuberculosis (TB), which is caused by persistent infection with the bacillus *Mycobacterium tuberculosis* (Mtb) (1). Despite advances in antibiotic drug regimens, completion of the prolonged therapy is challenging, and poor adherence is a major cause of treatment failure and the rise of multi-drug resistant strains (1). An efficient host immune response to the pathogen is critical to control Mtb infection (2, 3), with T cells being directly involved in limiting pathogen spread (4). However, Mtb has evolved complex mechanisms to not only evade immune detection but also to subvert the efficacy of host immunity and promote tolerogenic responses, thus allowing persistent infection in most individuals (5, 6). Consequently, Mtb-specific T cells often appear impaired in number and functional capacities during disease progression (7).

One strategy to address Mtb persistence in the face of ineffective host immune responses is to use T cell receptor (TCR) bispecific molecules targeting intracellular mycobacterial antigens to eliminate Mtb-infected cells via redirection of functional polyclonal T cells. TCR-based T cell redirecting molecules combine an affinity-enhanced TCR domain binding disease-associated peptide HLA (pHLA) complexes on the target cell surface and an anti-CD3 domain to recruit and activate multiple polyfunctional T cell subsets (8–11). Such molecules, capable of redirecting T cells toward cancer- or virus-associated pHLAs (Immune-mobilizing monoclonal TCR Against Cancer/Virus—ImmTAC and ImmTAV, respectively), have been previously developed and are currently under clinical evaluation for treatment of cancer and viral infections, thus providing rationale to exploit a similar strategy to target mycobacterial antigens generated within infected cells. Indeed, the first ImmTAC targeting an HLA-A*02:01-restricted peptide from gp100 has been recently approved for the treatment of uveal melanoma, providing overall survival benefit in patients (12).

The therapeutic potential shown by TCR-based approaches in clinical studies is fostering considerable efforts to further enhance their efficacy and broaden their applicability (13, 14). A major hurdle for all current TCR-based therapies is their restriction to classical HLA Ia molecules (15, 16), which are highly polymorphic across the human population. Consequently, only patients expressing specific HLA Ia allelic variants can benefit from these therapies. To circumvent this limitation in the context of mycobacterial infection, and potentially expand the number of treatable patients, we explored the concept of targeting mycobacterial peptides presented by nonclassical HLA Ib molecules, which are structurally very similar to classical HLA Ia molecules but display very limited polymorphism.

HLA-E is a member of the nonclassical HLA Ib family and in humans displays only two functional alleles that differ by a single amino acid located outside the peptide binding groove (17, 18). Under homeostatic conditions, HLA-E presents leader-derived peptides from other HLA proteins and acts as a ligand for the CD94/NKG2 receptor complexes, regulating natural killer (NK) cell function (19, 20). However, following cellular stress, such as during infection, HLA-E can also present diverse pathogen- or self-derived peptides, which can be recognized by specific T cells (21–26). HLA-E-restricted CD8^+^ T cells recognizing Mtb-derived peptides presented by HLA-E have been described, and their capacity to kill macrophages infected with Mtb or *Mycobacterium bovis* has been reported (27–30).

Here, we describe a TCR-based bispecific molecule that redirects polyclonal T cells to target Mtb-infected cells through recognition of an Mtb HLA-E-presented peptide. This ImmTAB (Immunemobilizing monoclonal TCR Against Bacteria) represents a treatment strategy for TB and provides proof of concept for the development of TCR-based therapies circumventing HLA polymorphism while still accessing the intracellular antigenic landscape.

## Results

### HLA-E Stably Binds and Presents a Peptide from the Mycobacterial Enoyl-Reductase.

**Fig. 1.**
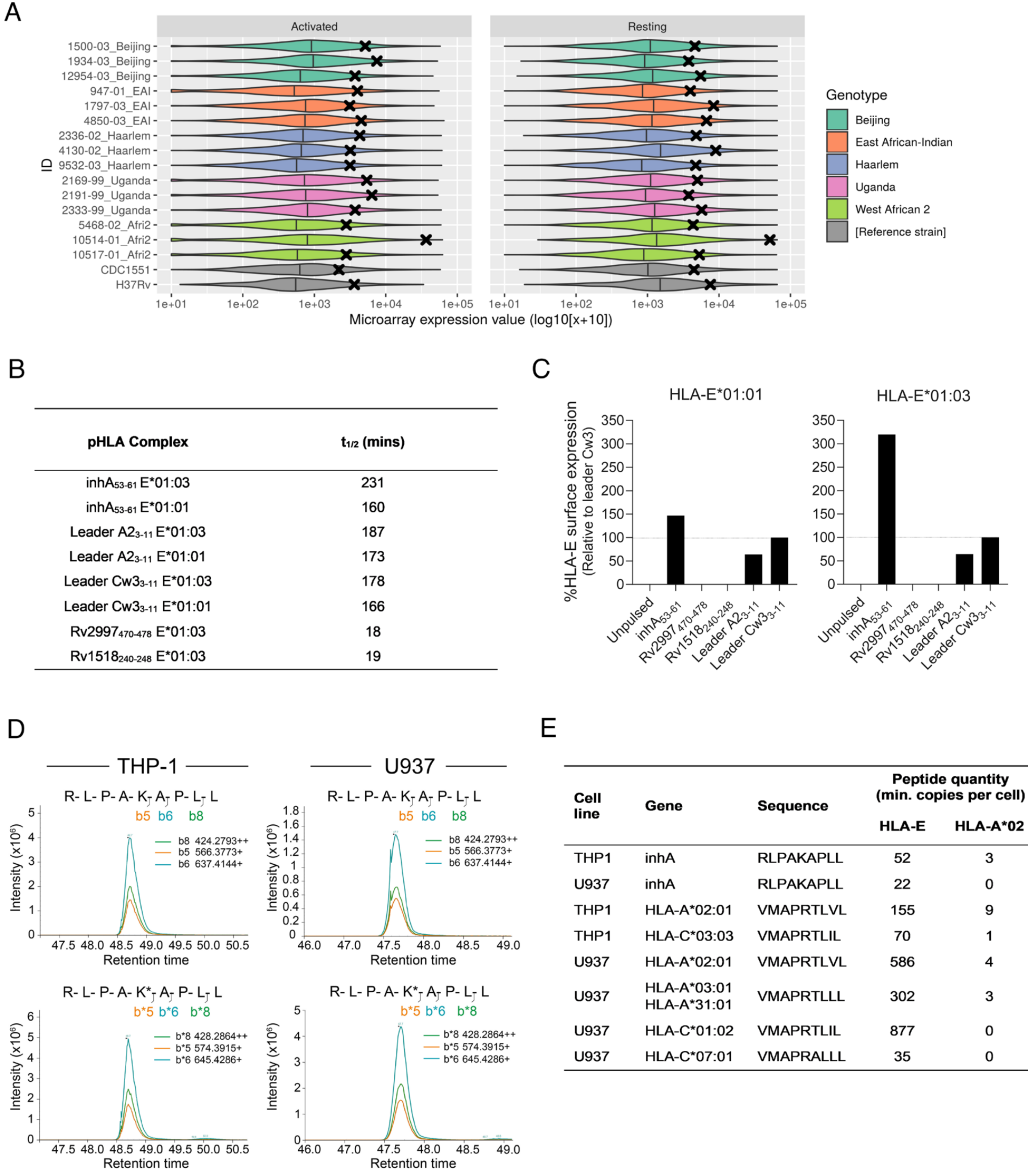
Identification and validation of a stable Mtb peptide in HLA-E. (*A*) Mtb gene expression distributions generated from microarray data. Violin plots of Mtb complex gene expression in 15 clinical samples including 3 strains of each of 5 genotypes and 2 reference strains in activated (*Left*) or resting (*Right*) macrophages. The expression value of *inhA* in each sample is denoted by a black cross. (*B*) Half-life stability for a range of pHLA complexes containing leader and Mtb peptides, measured by SPR time course analysis. (*C*) Flow cytometry analysis of HLA-E surface expression of K562-E*01:01 and K562-E*01:03 cells either unpulsed or pulsed with the indicated peptide, normalized to leader peptide Cw3_3-11_. (*D*) LC–MS quantitation of inhA_53-61_ in HLA-E and HLA-A*02:01 complexes immunopurified from THP-1 (*Left*) and U937 cells (*Right*). Extracted ion chromatograms for native (*Top* chromatograms) and spiked in synthetic isotopically labeled inhA_53-61_ peptides (*Bottom* chromatograms; marked with*) are shown. (*E*) Absolute quantities of indicated peptides (inhA_53-61_ and derived from HLA-leader) within immunopurified HLA-E.

We adopted a systematic approach to target selection, specifically searching for peptides derived from Mtb proteins encoded by abundantly expressed genes with high sequence conservation that could generate stable complexes with HLA-E. First, we performed a global assessment of the Mtb proteome to identify peptides predicted to bind HLA-E. The best candidate peptide was RLPAKAPLL, derived from mycobacterial enoyl reductase, encoded by the *inhA* gene at the locus Rv1484 (predicted IC_50_ = 35.3 nM). This peptide, herein referred to as inhA_53-61_, has previously been shown to elicit HLA-E restricted T cell responses in Mtb-infected donors (28), and T cell clones targeting this peptide can kill Mtb-infected cells (29, 30). Interrogation of *inhA* gene expression in a diverse group of clinically relevant samples revealed *inhA* to be in the top 7% of genes ranked by expression value across both Mtb-infected resting and activated macrophages (Fig. 1*A*). Furthermore, the Mtb genome variation resource, tbvar, identified no genomic variations in the region corresponding to inhA_53-61_ (locus Rv1484, genomic nucleotide coordinates 1674358 to 1674385) (31). This corroborated other findings that nonsynonymous mutations in *inhA* are rare and thus unlikely to affect peptide sequence (32).

We employed surface plasmon resonance (SPR) to assess binding of the inhA_53-61_ peptide to HLA-E and to determine the half-life of inhA_53-61_ in complex with soluble recombinant HLA-E molecules. We observed that inhA_53-61_ formed stable complexes with both HLA-E alleles (Fig. 1*B*), comparable to those measured for control HLA leader-derived peptides.

The ability of the inhA_53-61_ peptide to bind HLA-E was further tested in a cell pulsing assay. HLA-I-deficient K562 cells were transduced with beta-2-microglobulin (β_2_m)-HLA-E*01:01 or β_2_m-HLA-E*01:03 single chain gene-fusion constructs (K562-E*01:01 and K562-E*01:03, respectively), and pulsed with the inhA_53-61_ peptide, other Mtb peptides, or control peptides derived from leader regions of two HLA variants known to bind HLA-E (*SI Appendix*, Table S1). Cell surface HLA-E levels increased in the presence of inhA_53-61_ and the leader-derived peptides (Fig. 1*C*), suggesting peptide dependent binding to and stabilization of HLA-E molecules on the cell membrane. No increase in HLA-E surface levels was induced by the other Mtb peptides, previously reported to be recognized by HLA-E-restricted T cells (28). A greater inhA_53-61_-dependent increase in surface HLA-E relative to a control HLA-Cw3 leader-derived peptide was observed in the context of HLA-E*01:03 compared to HLA-E*01:01 (Fig. 1*C*), in agreement with an earlier report (33).

To investigate whether inhA_53-61_ could be processed and loaded on HLA-E within cells, we ectopically expressed the full *inhA* gene in two monocytic cell lines endogenously expressing HLA-E (U937 – HLA-E*01:01/*01:03 heterozygous; THP-1 – HLA-E*01:03 homozygous). These cell lines were also transduced to express β_2_m-HLA-A*02:01 to investigate possible presentation of inhA_53-61_ by HLA-A*02:01, as some HLA-E peptides have dual binding capacity (34). Additionally, as a peptide derived from the leader region of HLA-A*02:01 is a known and stable natural HLA-E ligand, its overexpression allowed us to assess whether inhA_53-61_ could compete with this HLA-A*02:01 leader-derived peptide for presentation on HLA-E.

Quantitative proteomics revealed that inhA_53-61_-HLA-E complexes were present on the surface of both cell lines at between 22 and 52 copies per cell, with little or no evidence of inhA_53-61_ presentation on HLA-A*02:01 (Fig. 1 *D* and *E*, *SI Appendix*, Fig. S1, and Dataset S1). Furthermore, simultaneous quantitation of different HLA leader-derived peptides in both cell lines showed that inhA_53-61_ was presented at levels falling within the lower end of the range observed for these known endogenous HLA-E ligands (35 to 877 copies per cell, Fig. 1*E*). Collectively, these data indicated that the inhA_53-61_ peptide is stably bound by HLA-E and presented in complex with HLA-E on the cell surface following the cellular processing of ectopically expressed mycobacterial enoyl reductase. Therefore, this peptide may represent a natural HLA-E ligand in Mtb-infected cells.

### Generation of a TCR Bispecific Molecule (ImmTAB-inhA) that Targets the Mycobacterial inhA_53-61_ Peptide in Complex with HLA-E.

**Fig. 2.**
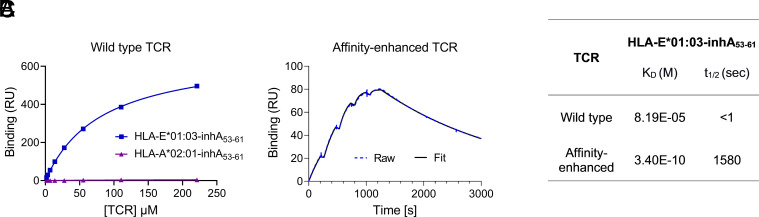
Binding of wild-type and affinity-enhanced TCRs to HLA-E*01:03-inhA_53-61_. (*A*) Steady-state analysis was used to measure affinity (K_D_) of the wild-type TCR to HLA-E*01:03-inhA_53-61_ complex using a serial dilution of TCR; t_1/2_ was calculated using the dissociation rate constant. Binding of the wild-type TCR to HLA-A*02:01-inhA_53-61_ complex was also assessed. (*B*) Single-cycle kinetics was used to assess binding (K_D_ and t_1/2_) of the affinity-enhanced a42b20 TCR to HLA-E*01:03-inhA_53-61_ complex. (*C*) Summary of K_D_ and t_1/2_ values of the TCRs, representative of two independent measurements.

Synthetic TCR libraries were screened to identify TCRs specific for the HLA-E-inhA_53-61_ complex. A TCR that exhibited binding to the inhA_53-61_ peptide in complex with both HLA-E alleles, but not to the inhA_53-61_ peptide complexed with HLA-A*02:01 (Fig. 2*A*), was isolated and subjected to iterative affinity enhancement. This process resulted in a TCR variant (a42b20) with increased binding affinity to HLA-E-inhA_53-61_, from 82 μM to 340 pM and extended half-life of interaction, from <1 s to 26 min (Fig. 2 *B* and *C*).

**Fig. 3.**
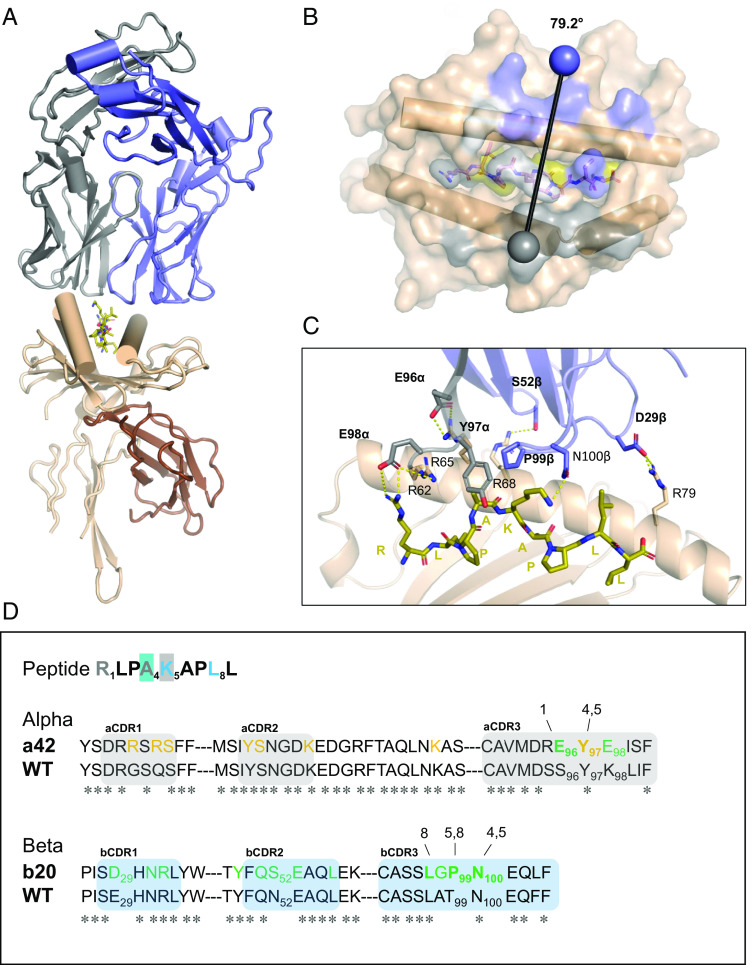
Structural overview of the affinity-enhanced a42b20 TCR in complex with HLA-E-inhA_53-61_ (*A*) Overall ribbon representation of TCR a42b20 in complex with HLA-E-inhA_53-61_; TCR alpha chain is colored gray, beta chain is blue, HLA chain is cream, the β_2_m is brown, and the peptide in olive stick representation. (*B*) *Top–down* view showing the pHLA surface; for orientation, HLA helices 1 and 2 are drawn as cylinders. pHLA surface is colored as in (*A*); residues that interact with the TCR alpha chain are colored gray, residues that interact with the TCR beta chain are in blue, and residues that interact with both the alpha and beta chains are in white. Gray and blue spheres show the position of the conserved disulfide bond in the alpha and beta variable domains, respectively; the vector joining them shows the crossing angle (79.2°). (*C*) Close-up view of the TCR-pHLA interface with residues introduced through affinity enhancement drawn as sticks and labeled in bold, hydrogen bonds and salt-bridges represented by yellow dashes. (*D*) TCR-pHLA interactions mapped onto sequence with truncated a42b20 and wild-type sequences provided to show sites modified by affinity enhancement. TCR alpha CDR loops are colored gray, whereas the beta CDR loops are colored blue. Peptide residues in gray or in blue font indicate that they are within 4.1 Å of TCR alpha chain or beta chain, respectively. Peptide residues 4 and 5 are highlighted in blue or in gray since they are also within 4.1 Å TCR of beta and alpha chain, respectively. TCR sequence annotation: positions in yellow or green font indicate that they are within 4.1 Å of HLA helix 2 or helix 1, respectively. Residues in bold indicate that they are within 4.1 Å of a peptide residue, with numbering above residues indicating the relevant peptide position/s.

The crystal structure of a42b20 TCR in complex with pHLA-E-inhA_53-61_ was determined at 2.33 Å resolution (*SI Appendix*, Table S2) and revealed a canonical binding mode (Fig. 3*A*), with a crossing angle of 79.2° and a large protein–protein interface. The TCR buries some 875 Å^2^ at the HLA-E interface and 243 Å^2^ at the peptide interface [calculated using PISA (35)] (Fig. 3*B*) and engages with both HLA-E helices and the peptide ligand (Fig. 3*C*). Interactions with the HLA-E helix 2 are made through the TCR alpha chain and those with the HLA-E helix 1 predominantly through the TCR beta chain. The TCR also interacts with all accessible peptide positions (R1, A4, K5, and L8), likely contributing to the observed specificity of the molecule.

Analysis of the a42b20 TCR-pHLA binding interface in the context of the wild-type sequence (Fig. 3*D*) revealed that the affinity enhancement process introduced new interactions to both HLA and peptide residues. Residues in the alpha and beta CDR3 loops are solely responsible for direct peptide interactions and those introduced during the engineering process include E96α, Y97α, and P99β. E96α forms a salt bridge with the lysine at the P1 position of the peptide, likely contributing to peptide sensitivity for that position (Fig. 3*D*). In addition, the CDR3 Y97α and CDR3 P99β residues flank peptide residues A4 and K5, forming hydrophobic contacts (Fig. 3 *C* and *D*). All CDR loops contribute to binding the HLA-E surface (Fig. 3*D*) and include several amino acids introduced during the affinity enhancement process. Residues E96α, E98α, S52β, and D29β make polar contacts to HLA-E residues R62, R65, R68, and R79, respectively (Fig. 3*C*).

These data show that the TCR selection and engineering process yielded an affinity-enhanced TCR that tightly binds HLA-E in complex with the inhA_53-61_ peptide, by establishing crucial stabilizing contacts with both the peptide and HLA-E itself. The a42b20 TCR was therefore fused to an anti-CD3 single-chain variable fragment domain (8, 9) to generate the bispecific molecule ImmTAB-inhA, which was then investigated for potency and specificity in multiple cellular assays.

### ImmTAB-inhA Specifically Redirects T Cells against Cells Expressing Both HLA-E and the Mycobacterial *inhA* Gene.

To test the ability of ImmTAB-inhA to redirect T cells against target cells presenting the cognate epitope on HLA-E*01:01 or -01:03, CRISPR/Cas9 technology was used to generate THP-1 cells deficient in β_2_m and CIITA gene expression (36, 37), and therefore lacking both classical HLAs (I and II) and HLA-like molecules on their surface. These cells were subsequently transduced with a single chain HLA-E-β2m gene-fusion construct encoding either HLA-E*01:01 or HLA-E*01:03 proteins (herein referred to as THP-1-E cells) to exclusively monitor HLA-E-dependent responses in the absence of any other classical or nonclassical HLA molecules. IFN-γ release by peripheral blood mononuclear cells (PBMC) from healthy donors in the presence of ImmTAB-inhA and THP-1-E cells pulsed with the target peptide was then monitored.

**Fig. 4.**
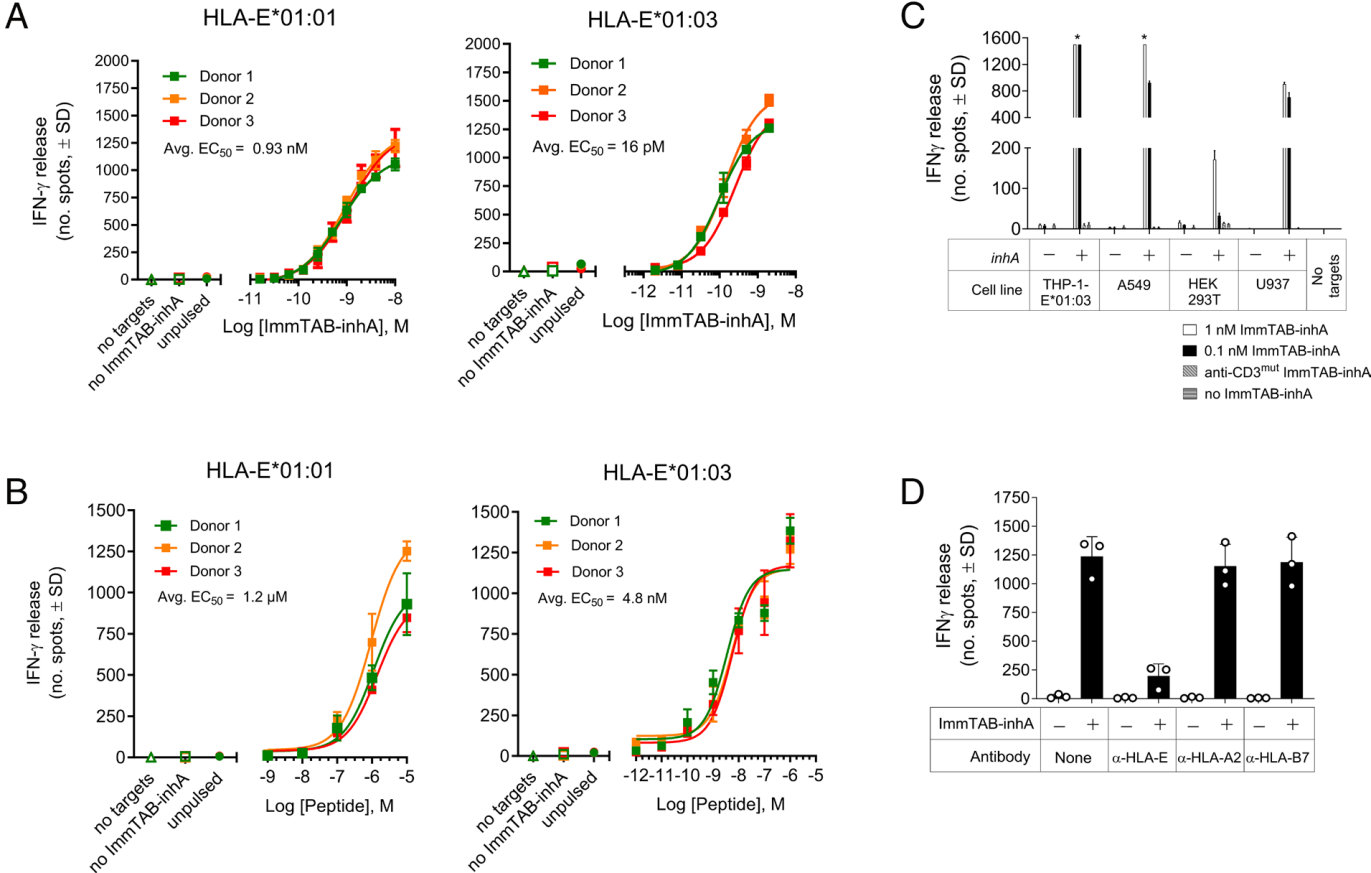
ImmTAB-inhA elicit T cell responses against cells displaying the cognate pHLA-E complexes. (*A*) ELISPOT of dose-dependent IFN-γ release induced by ImmTAB-inhA in cocultures of PBMC from three healthy donors and THP-1-E cells pulsed with inhA_53-61_ (10 μM). (*B*) IFN-γ ELISPOT assays showing titratable activation of PBMC from three healthy donors by ImmTAB-inhA (2 nM) in the presence of THP-1-E cells pulsed with titrated levels of peptide. Controls (*A* and *B*) include: PBMC+ImmTAB-inhA (no target), PBMC+target cells (no ImmTAB), and PBMC+ImmTAB-inhA+target cells (unpulsed). Data are plotted as mean ± SD of triplicates. (*C*) IFN-γ release from healthy donor PBMC in the presence of indicated cell lines transduced with full-length inhA (+) or untransduced controls (–). ImmTAB-inhA was added at concentrations of 1 nM (white bars) or 0.1 nM (black bars). No target, a CD3 nonbinding ImmTAB-inhA (anti-CD3^mut^ ImmTAB-inhA), and no ImmTAB-inhA were included as controls. (*D*) IFN-γ responses induced by 1 nM ImmTAB with PBMC cocultured with THP-1-E*01:03 pulsed with inhA_53-61_ (10 μg/mL) in the presence or absence of blocking mAbs against HLA-E, HLA-A2, or HLA-B7 (10 μg/mL). *, too numerous to count. (*C* and *D*) are representative of one of three donors tested in triplicate.

ImmTAB-inhA dose-dependent IFN-γ secretion was detected against THP-1-E cells expressing either HLA-E allele in the presence of a fixed dose of the inhA_53-61_ peptide, with EC_50_ values of 0.93 nM for HLA-E*01:01 and 0.0016 nM for HLA-E*01:03 (Fig. 4*A*). In peptide titration experiments the ImmTAB-inhA-dependent IFN-γ response occurred at low peptide concentrations (Fig. 4*B*), albeit with a difference in potency observed between HLA-E*01:01 and HLA-E*01:03 (EC_50_ = 1.2 mM and 4.8 nM, respectively), possibly due to the different stability of the complexes formed by the peptide with the two individual allelic variants (Fig. 1 *B* and *C*). ImmTAB-inhA also mediated specific IFN-γ secretion by healthy donor PBMC against multiple cell lines, either overexpressing HLA-E (THP-1-E) or displaying endogenous HLA-E (A549, HEK293T and U937 cells), when transduced with the full *inhA* gene (antigen positive; Ag+) and thus endogenously processing the target peptide (Fig. 4*C*). No IFN-γ release was detected against nontransduced cells (antigen negative; Ag−) or when a modified version of ImmTAB-inhA, containing a mutated anti-CD3 domain unable to engage CD3 on T cells (anti-CD3^mut^ ImmTAB-inhA), was used against either *inhA*-transduced or nontransduced cells (Fig. 4*C*). Furthermore, ImmTAB-inhA-dependent IFN-γ release against THP-1-E cells pulsed with the target peptide was inhibited in the presence of anti-HLA-E blocking mAbs (Fig. 4*D*), confirming HLA-E-restriction and specificity of ImmTAB-inhA. These data highlighted the capacity of ImmTAB-inhA to specifically redirect T cells toward cells presenting the inhA_53-61_ peptide, either exogenously provided or endogenously processed, in complex with HLA-E.

To further test the selectivity of ImmTAB-inhA, THP-1-E cells pulsed with different leader-derived peptides from multiple HLA alleles, a range of potential mimetic peptides from the human proteome (defined in materials and methods) or previously described peptide ligands from other microbes (*SI Appendix*, Table S1) were tested for their capacity to trigger ImmTAB-inhA-mediated responses. Apart from the control cognate inhA_53-61_ peptide, none of the peptides tested were able to elicit IFN-γ release by healthy donor PBMC in the presence of ImmTAB-inhA (*SI Appendix*, Fig. S2). Furthermore, in parallel experiments, ImmTAB-inhA molecules did not induce IFN-γ secretion by PBMC in response to a panel of inhA antigen negative cancer cell lines expressing various levels of HLA-E and presenting different sets of endogenous HLA-E ligands. Finally, ImmTAB-inhA molecules did not elicit any PBMC reactivity against a panel of normal cells representing vital tissues (colon, intestine, heart, kidney, lung, and brain) (*SI Appendix*, Figs. S2 and S3 and Tables S3 and S4). The selectivity and specificity of ImmTAB-inhA were further tested using TAP-deficient RMA-S cells transfected with HLA-E (RMA-S/HLA-E), as inhA_53-61_ peptide has been previously shown to bind to and stabilize HLA-E molecules on the surface of RMA-S/HLA-E cells (27). In agreement with previous findings, we observed cell surface stabilization of HLA-E molecules by inhA_53-61_ (*SI Appendix*, Fig. S4). InhA_53-61_ peptide-pulsed RMA-S/HLA-E cells elicited IFN-γ and TNF-α expression by healthy donor PBMC CD3^+^ T cells in the presence of ImmTAB-inhA, while RMA-S/HLA-E cells that had been pulsed with a CMV UL40-derived peptide also known to bind to HLA-E, did not induce IFN-γ and TNF-α expression by CD3^+^ T cells in the presence of ImmTAB-inhA (*SI Appendix*, Fig. S5). Moreover, IFN-γ and TNF-α expression were inhibited by blocking mAbs to CD3 and HLA-E. Finally, inhA_53-61_ peptide-pulsed RMA-S/HLA-E cells elicited ImmTAB-inhA-dependent IFN-γ and TNF-α expression by CD8^+^, CD4^+^ or double-negative (DN) CD3^+^ T cells and in all instances, cytokine expression was inhibited by blocking mAbs to CD3 and HLA-E (*SI Appendix*, Fig. S5). Collectively, these data demonstrated the specificity of ImmTAB-inhA for HLA-E-inhA_53-61_ complexes and the lack of cross-reactivity toward any other HLA-E-presented peptide or HLA-E-expressing cell line tested.

### ImmTAB-inhA Mediates Activation of Multiple T Cell Subsets to Elicit Polyfunctional Responses.

**Fig. 5.**
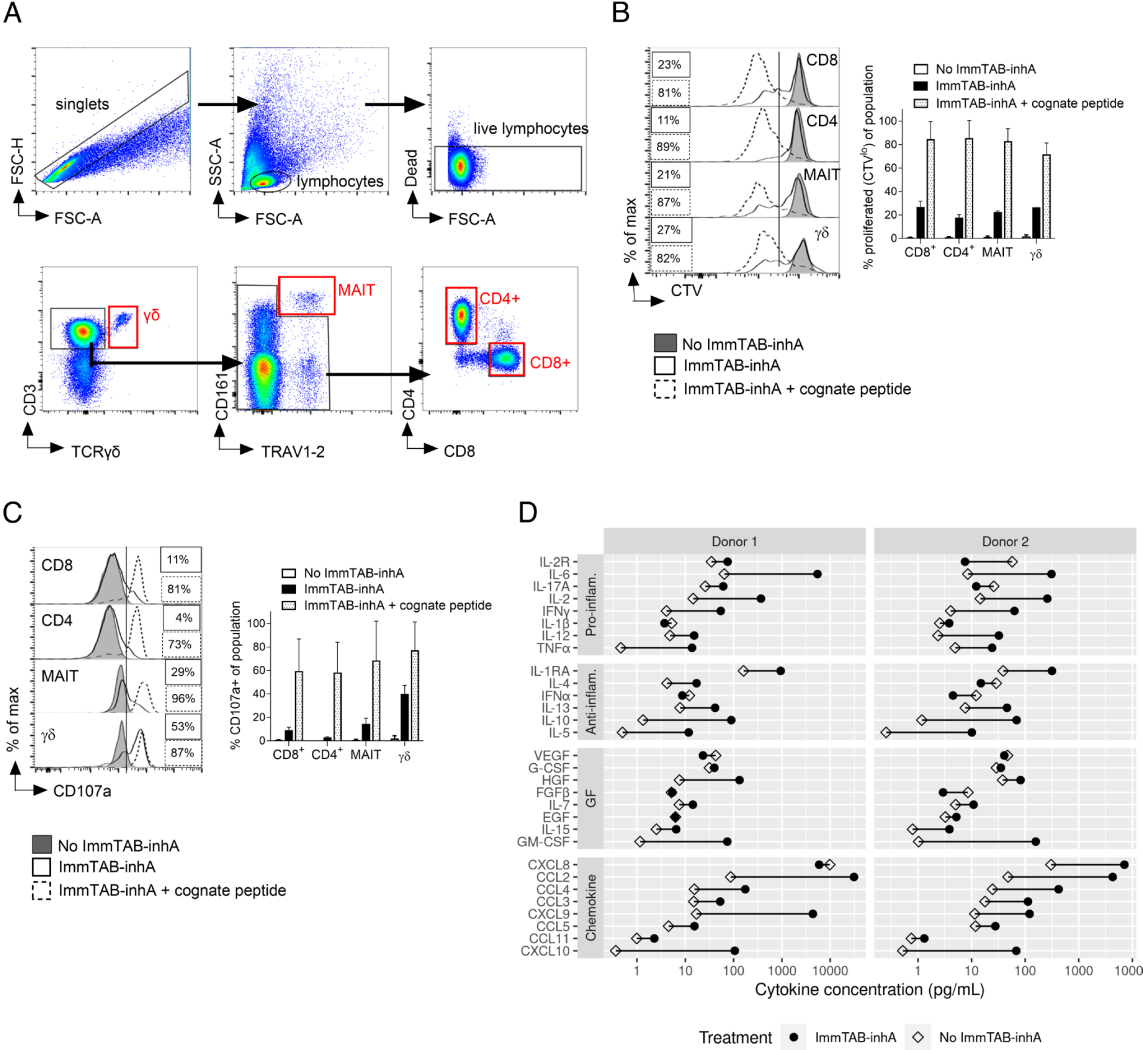
ImmTAB-inhA redirects multiple T cell subsets to elicit polyfunctional responses. (*A*) Gating strategy for identification of T cell subsets. T cell subsets were identified by gating first on singlets (FSC-A vs. FSC-H) and then lymphocytes (FSC-A vs. SSC-A). Live lymphocytes were then gated as cells excluding the fixable viability dye. From this negative gate, γδ T cells were identified as CD3^+^TCRγδ^+^. From the CD3^+^ TCRγδ^−^ gate, MAIT cells were identified as CD161^+^TRAV1-2^+^. The remaining cells were divided into CD4^+^CD8^−^ (CD4^+^ T cells) or CD4^−^CD8^+^ (CD8^+^ T cells). (*B* and *C*) Flow cytometry analysis of (*B*) CTV dilution and (*C*) CD107a^+^ surface levels induced by 1 nM ImmTAB-inhA in indicated T cell subsets identified by specific gating within PBMC cocultured with *inhA*-transduced HEK293T cells. The same analysis was performed within parallel control cocultures not treated with ImmTAB-inhA (no ImmTAB-inhA) or supplemented with ImmTAB-inhA and the cognate peptide (ImmTAB-inhA + cognate peptide). *Left* panels show representative histogram plots. *Right* panels depict cumulative results from four donors. Data in the *Right* panels are plotted as mean ± SD. (*D*) ImmTAB-inhA induces production of proinflammatory cytokines. PBMC effectors (E) were incubated with Ag+ HEK 293T target cells (T) at a 10:1 ratio in the absence or presence of 1 nM ImmTAB-inhA (I) for 5 d. Production of cytokines (proinflammatory and anti-inflammatory), GF, and chemokines was assessed in culture supernatants by the Luminex assay. The concentration of each cytokine is plotted for cultures without (E+T; open diamonds) and with ImmTAB-inhA (E+T+I; black circles) for each donor tested (n = 2).

To determine whether ImmTAB-inhA could redirect circulating effector T cell subpopulations of healthy donors toward *inhA*-expressing cells, PBMC were cocultured with *inhA*-transduced HEK293T cells endogenously expressing HLA-E in the presence or absence of ImmTAB-inhA. As positive control, *inhA*-transduced HEK293T cells were also pulsed with inhA_53-61_ peptide to increase the pHLA-E density on the surface of target cells. Flow cytometry was used to investigate four major T cell subsets, including both conventional CD8^+^ and CD4^+^ T cells and the innate-like mucosal-associated invariant T (MAIT) and γδ T cells, identified by specific gating (Fig. 5*A*). In the presence of peptide-pulsed target cells, all populations mounted potent ImmTAB-inhA-dependent proliferation and degranulation responses, as measured by Cell Trace Violet dilution (Fig. 5*B*) and CD107a surface staining (Fig. 5*C*), respectively. When antigen-transduced target cells were not pulsed with the peptide, responses were still detected, albeit to a lower extent, reflecting reduced epitope density on the target cell surface. As expected, despite all T cell subsets displaying proliferative responses against antigen-transduced targets, CD8^+^, MAIT, and γδ T cells showed enhanced degranulation as compared to CD4^+^ T cells, with MAIT and, in particular, γδ T cells being the most responsive (Fig. 5*C*), in line with the unique innate-like features of these two T cell subsets (38–42). Luminex analysis of the coculture supernatants also revealed that ImmTAB-inhA induced a significant increase in the secretion of multiple proinflammatory cytokines, anti-inflammatory mediators, growth factors (GF), and chemokines (Fig. 5*D*). These results demonstrated the ability of ImmTAB-inhA to efficiently stimulate polyfunctional effector responses against cells presenting its target pHLA-E.

### ImmTAB-inhA Mediates Specific T Cell Killing of Cells Expressing Both HLA-E and Mycobacterial inhA Gene.

**Fig. 6.**
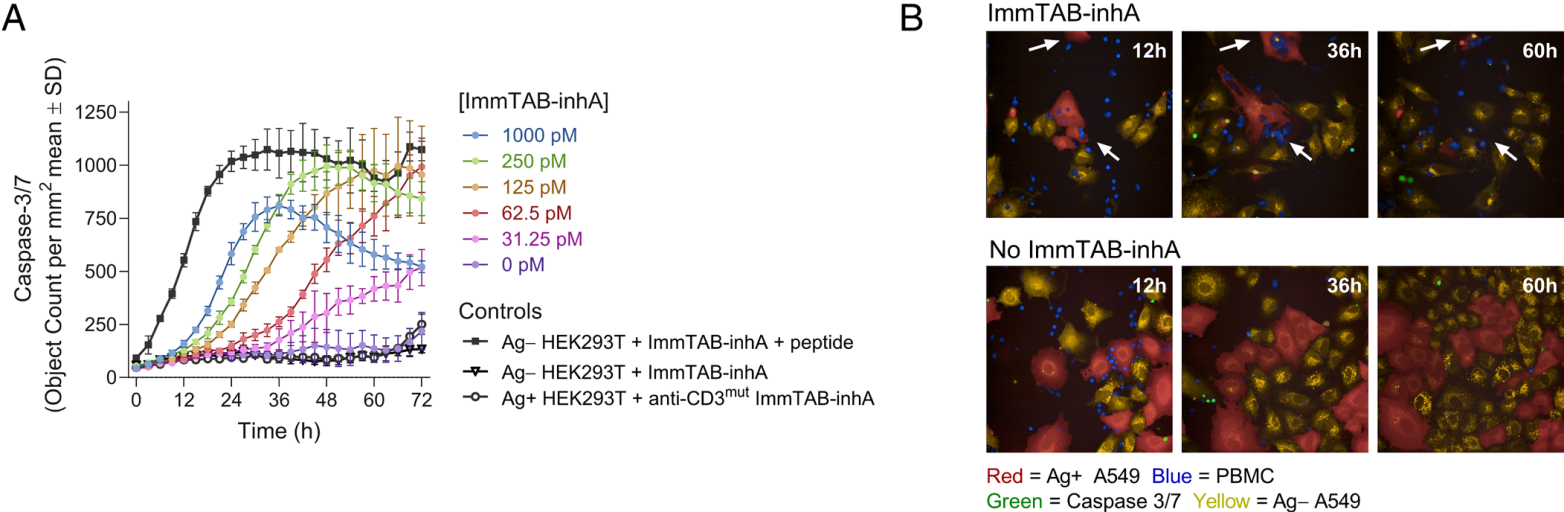
ImmTAB-inhA mediates antigen-dependent T cell killing of target cells. (*A*) Apoptosis of Ag+ HEK293T cells cocultured with PBMC and a titration of ImmTAB-inhA measured by IncuCyte. Controls include Ag+ HEK 293T in the presence of ImmTAB-inhA with or without cognate peptide and Ag- HEK 293T in the presence of a CD3 nonbinding version of ImmTAB-inhA (anti-CD3^mut^ ImmTAB-inhA). Data are representative from one of three donors tested in triplicate. (*B*) Confocal imaging of PBMC (blue) cocultured with a mixture of Ag+ (red; transduced with full-length *inhA*) and Ag− (yellow; nontransduced) A549 cells in the presence or absence of ImmTAB-inhA corresponding to Movies S1 and S2, respectively. Arrows indicate apoptosis of Ag+ target cells.

Next, we explored the potential of ImmTAB-inhA to mediate T cell killing of cells presenting the cognate target. In the presence of ImmTAB-inhA, we detected a dose-dependent lysis of inhA-transduced HEK293T cells by healthy donor PBMC, as measured by caspase-3/7 activation in target cells over time (Fig. 6*A*). Killing was observed from 12 h of coculture and, unless cognate peptide was added, no cytolysis of wild-type HEK293T cells lacking *inhA* expression was detected.

These results were confirmed and extended in a second cellular model using live cell imaging. In these experiments, *inhA*-expressing (Ag+) and nonexpressing (Ag−) lung epithelial A549 cells were mixed within the same wells before addition of ImmTAB-inhA and PBMC from healthy donors. In the presence of ImmTAB-inhA, only killing of Ag+ cells was observed, while Ag- cells remained viable (Fig. 6*B* and Movies S1 and S2). Taken together, these results demonstrated selective ImmTAB-inhA-mediated killing of Ag+ cells and lack of bystander killing of Ag- cells.

**Fig. 7.**
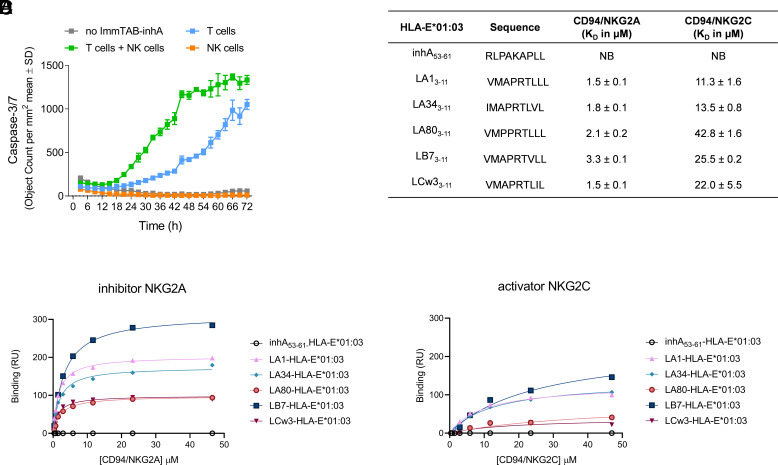
ImmTAB-inhA does not directly engage NK cells. (*A*) ImmTAB-inhA-mediated apoptosis of Ag+ HEK293T cells cultured with purified T cells, NK cells, or a mixture of the two populations measured by IncuCyte. Data represent mean ± SD of triplicates and are representative of four independent experiments. (*B*–*D*) Determination of affinities parameters for CD94/NKG2A/C–pHLA-E complex interactions by SPR. (*B*) Binding affinity K_D_ of CD94/NKG2A/C receptors for the panel of pHLA-E complexes analyzed; NB = no binding. Steady-state analysis was used to measure K_D_ affinity of CD94/NKG2A (*C*) and CD94/NKG2C (*D*) to pHLA-E complexes (inhA_53-61_ and leader peptides) using a serial dilution of CD94/NKG2A/C receptors.

As NK cells express both activating and inhibitory CD94/NKG2 receptors that bind HLA-E, we next investigated whether ImmTAB-inhA binding to HLA-E-inhA_53-61_ complexes on target cells could directly influence NK cell activity. Purified NK cells failed to kill *inhA*-transduced HEK293T (Ag+) cells in the presence of ImmTAB-inhA (Fig. 7*A*), indicating that ImmTAB-inhA did not directly engage and redirect NK cells. However, when purified NK cells were added to autologous T cells, a substantial ImmTAB-inhA-dependent increase in the cytotoxic response was observed as compared to T cells alone (Fig. 7*A*). Importantly, soluble forms of the CD94-NKG2A and -NKG2C NK cell receptor ectodomains failed to bind HLA-E-inhA_53-61_ complexes, as measured by SPR (Fig. 7 *B*–*D*). These data suggested that ImmTAB-inhA is unlikely to have a direct impact on NK cell functions mediated by the two NK receptors. Thus, the observed additive effect of NK cells on ImmTAB-inhA-induced cytolytic response could be due to their bystander stimulation following T cell activation.

### ImmTAB-inhA Redirects T Cells to Kill Mtb-Infected Primary Human Cells.

**Fig. 8.**
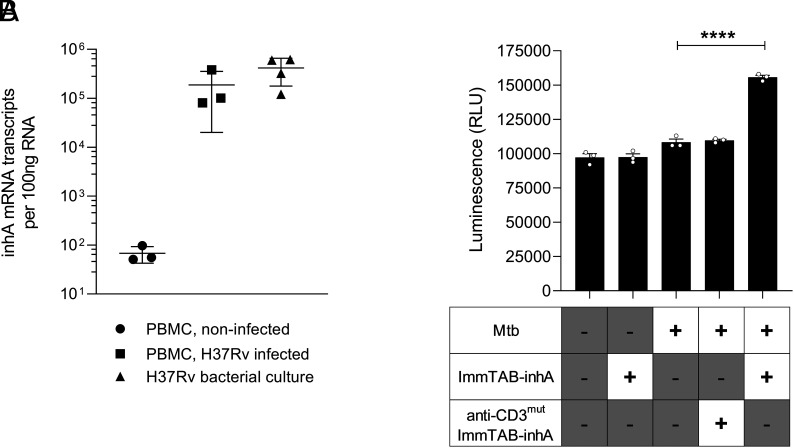
ImmTAB-inhA redirects T cells to kill Mtb-infected primary human cells. (*A*) Quantification of inhA mRNA in Mtb-infected PBMC by quantitative reverse transcription PCR plotting the average transcript number per 100 ng RNA from three PBMC donors. Mtb H37Rv cultured in broth and noninfected PBMC were included as positive and negative controls, respectively. (*B*) ImmTAB-inhA-mediated cell death of Mtb-infected primary cells in coculture with autologous healthy donor PBMC and 10 nM ImmTAB-inhA was determined by measuring luminescence using the ToxiLight assay. Controls included uninfected PBMC with and without ImmTAB-inhA, and Mtb-infected PBMC cocultured with or without a CD3 nonbinding version of ImmTAB-inhA (anti-CD3^mut^ ImmTAB-inhA). Data represent mean ± SD of triplicates and are representative of three healthy donor PBMC assayed. ****p < 0.01.

Finally, to assess whether ImmTAB-inhA could mediate killing of primary Mtb-infected human cells, PBMC from healthy donors were infected with virulent Mtb (H37Rv) at a multiplicity of infection (MOI) of 0.1. In these assays, bacilli are phagocytosed primarily by monocytes, resulting in ~25% of these cells becoming infected (10% of total PBMC) (43). Expression of the mycobacterial *inhA* gene in the cultures was confirmed 48 h postinfection by qPCR following extensive washing (Fig. 8*A*). ImmTAB-inhA, but not anti-CD3^mut^ ImmTAB-inhA, induced a significant increase in cell death within Mtb-infected cultures (Fig. 8*B*). These data indicated ImmTAB-inhA can mediate T cell–dependent elimination of primary human cells infected with Mtb.

### ImmTAB-inhA-Mediated Growth Inhibition of Intracellular Mtb.

To assess whether ImmTAB-inhA could also elicit growth inhibition of intracellular bacilli, human monocytic THP-1 cells were infected with virulent Mtb H37Rv at a MOI of 2 (i.e., 2 bacilli per macrophage) and used as targets of PBMC in the presence of ImmTAB-inhA. Having determined the optimal PBMC/THP-1 ratio, ImmTAB-inhA concentration, and time course, using a cohort of 41 PBMC donors, we observed an average 25% reduction of intracellular Mtb CFU, as compared to the respective controls (*SI Appendix*, Table S5).

**Fig. 9.**
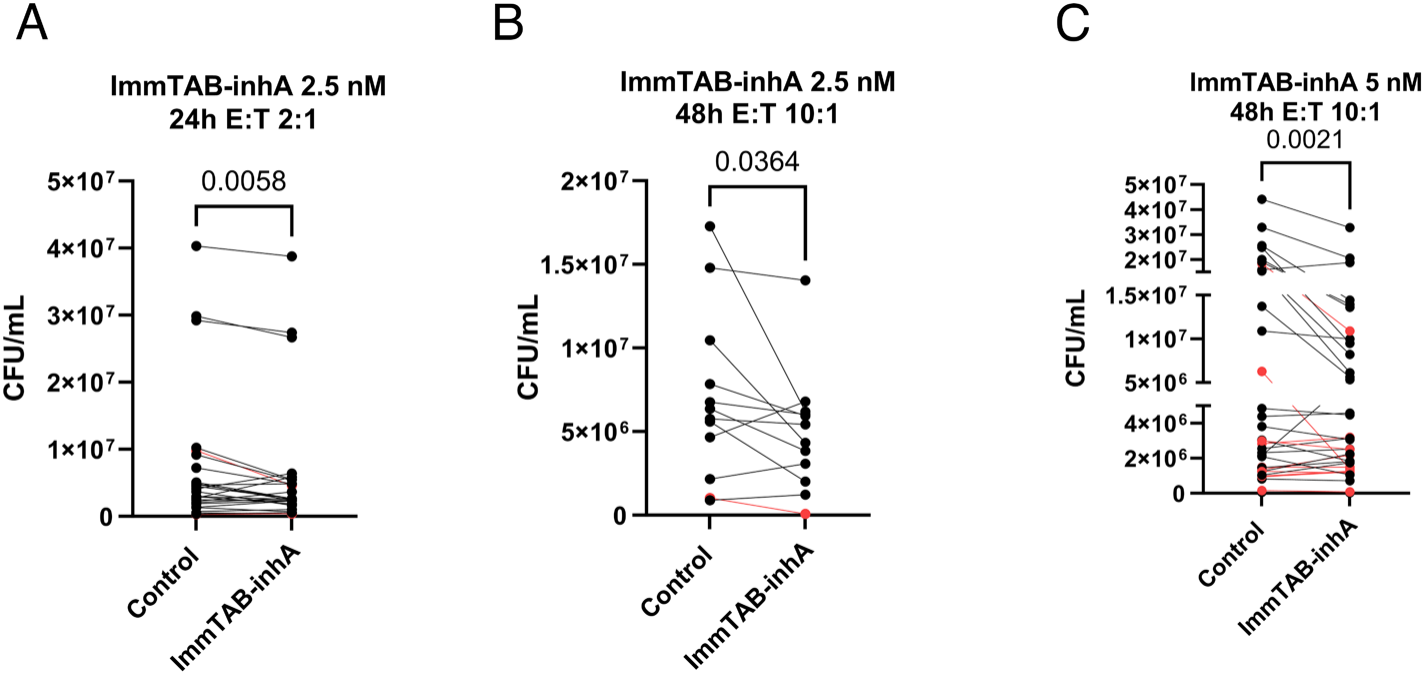
ImmTAB-inhA redirects PBMC to inhibit the viability of intracellular Mtb. Reduction of Mtb CFUs in three different experimental coculture conditions: (*A*) 2 × 10^5^ PBMC, 2.5 nM ImmTAB-inhA, 24 h incubation (n = 28); (*B*) 1 × 10^6^ PBMC, 2.5 nM ImmTAB-inhA, 48 h incubation (n = 12); (*C*) 5 × 10^5^ PBMC, 5 nM ImmTAB-inhA, 48 h incubation (n = 33). Responses from IGRA positive donors are shown in red. Paired *t-* test was used to assess whether the differences between experimental conditions were statistically significant.

Specifically, 2.5 nM ImmTAB-inhA induced a 15% reduction of Mtb CFU at 24 h, with E:T 2:1 (Fig. 9*A*) and 29% CFU reduction at 48 h with E:T 10:1 (Fig. 9*B*) while 5 nM ImmTAB-inhA induced a 31% reduction of Mtb CFU at 48 h with E:T 10:1 (Fig. 9*C*). In all three experimental conditions, the ImmTAB-inhA-mediated inhibition of intracellular Mtb growth was statistically significant. Of note, 32 out of the 41 tested PBMC samples exhibited a Mtb CFU reduction in the presence of ImmTAB-inhA in at least one experimental condition (Dataset S2). Eight out of forty-one PBMC donors were positive for the IGRA test indicating Mtb exposure, yet their baseline CFU reduction did not differ from that of unexposed donors. The responses observed with these donors against infected target are highlighted in red (Fig. 9 *A*–*C*).

### ImmTAB-inhA Enhances the Antimycobacterial Activity of Isoniazid and Ethambutol.

Next, we tested whether ImmTAB-inhA enhances the effect of two antibiotics against intracellular Mtb. Isoniazid and ethambutol are commonly used as first-line therapy against Mtb. To this aim, we cultured THP-1-derived macrophages that had been infected with Mtb at MOI 2 with 2.5 nM ImmTAB-inhA and PBMC from healthy donors (2 × 10^5^) in the absence or presence with isoniazid and ethambutol at three different concentrations [high (0.1 µg/mL isoniazid and 5 µg/mL ethambutol), intermediate (0.05 µg/mL isoniazid and 1 µg/mL ethambutol), and low (0.01 µg/mL isoniazid and 0.5 µg/mL ethambutol)]. We assessed Mtb CFU after 48 h incubation at 37 °C and 5% CO_2_. To normalize across experiments, results are shown as a percentage of CFU clearance compared to untreated Mtb-infected THP-1-derived macrophages set as 100%.

**Fig. 10.**
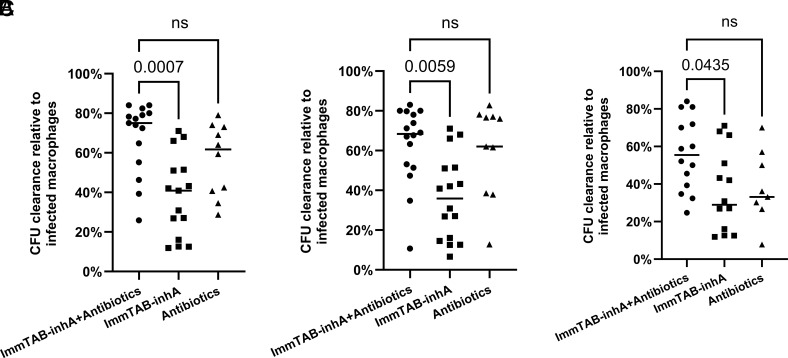
Combination of ImmTAB-inhA with isoniazid and ethambutol to inhibit the viability of intracellular Mtb. Mtb CFU measurements in Mtb-infected THP1 macrophages cocultured with PBMC (2 × 10^5^) and ImmTAB-inhA (2.5 nM) in the presence or absence of isoniazid and ethambutol at three different concentrations: (*A*) Isoniazid 0.1 µg/mL, ethambutol 5 µg/mL (n = 15); (*B*) Isoniazid 0.05 µg/mL, ethambutol 1 µg/mL (n = 16); (*C*) Isoniazid 0.01 µg/mL and ethambutol 0.5 µg/mL (n = 14). ImmTAB-inhA plus antibiotics (●), ImmTAB-inhA alone (■) and antibiotics alone (▲). Lines in each group represent the median. Kruskal-Wallis test was used to assess whether the differences between groups were statistically significant: ns, not significant.

ImmTAB-inhA elicited a 36% reduction of Mtb CFU on average and antibiotics yielded an average 48% reduction of Mtb CFU counts when tested independently (Fig. 10 *A*–*C*). Addition of ImmTAB-inhA moderately improved the efficacy of antibiotic treatment, at all tested antibiotic concentrations, although the difference was not statistically significant. Specifically, the combination of high dose antibiotics and ImmTAB-inhA reduced the CFU by 68% (Fig. 10*A*), the combination of intermediate dose antibiotics and ImmTAB-inhA reduced the CFU by 63% (Fig. 10*B*) and the combination of low dose antibiotics and ImmTAB-inhA reduced the CFU by 56% (Fig. 10*C*). These results highlight the potential benefit in combining ImmTAB-inhA with isoniazid and ethambutol to effectively eliminate intracellular Mtb.

## Discussion

HLA-E can present noncanonical peptides derived from pathogen-associated, stress-related, or normal proteins in conditions of infection, cell stress, or defective antigen-processing and presentation machinery (44–47). Noncanonical peptides display higher sequence heterogeneity as compared to classical HLA-leader-derived peptides presented by HLA-E, can be recognized by specific T cells through their TCR, and have been identified in the context of various infectious diseases and potentially cancers (46, 48). However, so far it has not been explored whether these peptides can serve as therapeutic targets for broadly applicable TCR-based approaches exploiting the very limited polymorphism of HLA-E.

Here, we generated a TCR-based bispecific molecule (ImmTAB-inhA) that targets an HLA-E-presented peptide derived from the enoyl reductase of Mtb, which has been previously reported to stimulate specific HLA-E-restricted T cells from infected and healthy individuals (27, 28, 49). ImmTAB-inhA molecules showed high potency and remarkable specificity in a range of biophysical assays and functional T cell redirection experiments. Importantly, potency and specificity were achieved by the introduction of multiple structural modifications in the TCR targeting domain through an engineering process, which generated new contacts with both the peptide and HLA-E, as revealed by sequence alignment and structural analyses. The high-affinity TCR component of ImmTAB-inhA can detect low copy numbers of cell surface pHLA-E complexes and thus has the potential to target cells with low bacterial burden opening up additional therapeutic avenues, such as chemoprophylaxis for infected individuals or allowing treatment shortening after the intensive phase of antibiotic therapy (50, 51).

TB still claims over 1.6 million lives annually, despite the existence of established diagnostic, chemotherapy, and BCG vaccination protocols (52). Although innate and adaptive T cell–mediated immune responses confer protection to approximately 90% of those infected, they fall short of eradicating the infection (51). Mtb is an intracellular pathogen capable of persisting within macrophages and evading the host immune system, making it difficult to treat (2). Mtb establishes a persistent intracellular presence within macrophages by impeding phagosome maturation and inhibiting phagolysosome fusion, which is essential for eliminating bacterial pathogens (53). Our data suggest that ImmTAB-inhA can effectively target an intracellular peptide presented by HLA-E on the surface of cells infected by Mtb, providing an approach to overcome pathogen resistance to T cell immunity. In fact, by redirecting polyclonal T cells toward Mtb-infected cells, ImmTAB-inhA offers a strategy to boost host T cell responses against Mtb while bypassing the need for activation of the Mtb-specific T cell pool, which is often impaired in late-stage infection (54). A target-specific and polyfunctional response was induced by ImmTAB-inhA in vitro in circulating cells of healthy donors, driven by the activation of multiple T cell subsets including both conventional and innate-like T cells. The activation of multiple T cell subsets leads to the production of IFN-γ and TNF-α which exert a protective role through the activation of macrophages (55, 56). Macrophage activation, which is an essential step for combating Mtb infection, promotes production of reactive nitrogen and oxygen species that have been shown to inhibit Mtb growth. In addition, macrophage activation also promotes upregulation of MHC molecules that most likely will result in an increase of the number of targetable HLA-E-inhA_53-61_ molecules (57–61). The recruitment and activation of MAIT and γδ T cells by ImmTAB-inhA may possibly have an important role in the elimination of Mtb-infected cells due to their recognized effector capacities. In fact, these innate-like T cells have recently raised great interest within the scientific community as potential tools for immunotherapy of TB because of their emerging importance in the host response to Mtb (62–64).

Taken together, our data suggest the possibility for ImmTAB-inhA molecules to recruit multiple T cell subpopulations with broad effector capacities to the sites of infection in TB patients and therefore induce local antimicrobial immune-mediated functions including the elimination of Mtb-infected cells (6) and the T cell–dependent activation of phagocytic cells (65). Thus, ImmTAB-inhA may facilitate T cell–mediated clearance of intracellular bacteria as well as the release of the mycobacteria from their intracellular niche, which could make them more susceptible to standard antibiotic treatments. In the present study, the combination of ImmTAB-inhA with two antibiotics used as first-line therapy against Mtb (isoniazid and ethambutol) resulted in only moderate improvement in the efficacy of either modality alone when tested against intracellular Mtb, possibly as a consequence of short-term culture. In a clinical setting, there is potential for a prolonged impact on persistent Mtb with a multiple dose regimen. An additional consideration is that isoniazid, a bactericidal agent, is only effective against metabolically active Mtb and may be rendered inactive due to common mutations in katG, inhA or its promoter region (66–68). Interestingly, mutations in the promoter region of inhA lead to overexpression of inhA itself (69–71), potentially increasing HLA-E-inhA_53-61_ complexes available at the cell surface for targeting infected cells with ImmTAB-inhA molecules. This drug resistance mechanism could therefore render Mtb-infected cells more susceptible to our TCR-based T cell redirecting therapeutic approach when used in combination with isoniazid, although it warrants further investigation. Thus, as ImmTAB-inhA represents a host-directed approach for Mtb therapy, it may circumvent established antimicrobial resistance mechanisms (66, 72, 73).

It is of note that we observed enhanced ImmTAB-inhA-induced cytotoxic responses in vitro in the presence of NK cells, despite not detecting any direct effects of ImmTAB-inhA on these cells, nor binding of the two major NK cell receptors to HLA-E-inhA_53-61_ complexes. These data suggested that ImmTAB-inhA molecules are unlikely to directly interfere with normal NK cell functions or compete with NK receptors for binding to their target, which might otherwise limit ImmTAB-inhA therapeutic efficacy. It is conceivable to interpret the additive effect of NK cells on the killing response as a likely result of their bystander stimulation following T cell activation in vitro. Whether this might occur in vivo warrants further investigation.

We postulate that the ImmTAB-inhA immunotherapy approach offers a potential solution for preventing active TB in people living with HIV (PLWH). The risk of TB is increased in PLWH, even before CD4^+^ T cell counts decline significantly (74). Furthermore, active vaccination with BCG is contraindicated in PLWH due to the risk of dissemination (75, 76), and combining antiretroviral regiments with TB chemotherapy can be challenging due to potential drug-drug interaction (77). Given the challenges of current treatment regimens and the lack of an effective prophylactic vaccine, exploring alternatives for TB control is crucial (78). In coinfected individuals, HLA-E expression remains intact as HIV-1 does not downmodulate it as it does with MHC class Ia molecules (79, 80). Immunoprophylaxis using ImmTAB-inhA could be a key strategy for this at-risk population, providing a unique approach to disease prevention (81, 82).

In conclusion, our study revealed the feasibility of specifically redirecting T cell responses toward a mycobacterial antigen presented by HLA-E as an additional treatment for TB on its own or in combination with antibiotics commonly used as first-line therapy. Furthermore, it provides proof-of-principle evidence that pathogen-derived peptides in complex with HLA-E can function as targets for TCR-based immunotherapies, thus circumventing classical HLA class I restriction while retaining the ability to access the intracellular antigenic landscape.

## Materials and Methods

### Cells.

Effector cells were isolated from whole blood obtained from anonymized healthy volunteers who consented to donate at Immunocore as part of a UK Health Research Authority–approved study. The study protocol (REC reference 13/SC/0226) was approved by the Oxford A Research Ethics Committee. Briefly, PBMC were isolated by density centrifugation using Ficoll-Hypaque. Pan T and NK cells were isolated from PBMC by negative selection (Miltenyi Biotec, Germany). THP-1 cells were genetically modified using CRISPR-Cas9 nickase to eliminate endogenous β_2_m and CIITA proteins. To generate cell lines ectopically expressing HLA-A*02:01/β_2_m (A2B2M), HLA-E, and/or Mtb antigen inhA, plasmids were designed and cloned for use in lentiviral transductions. See *SI Appendix* for details.

### Mtb Peptides and Human Peptides Homologous to Mtb Target.

To identify peptides that are potential HLA-E binders, we passed the entire proteome of the H37Rv reference sequence of Mtb through the netMHCpan4.0 peptide-HLA binding prediction algorithm (83). To minimize the risk of off-target activity, the entire human proteome (UniProt reference UP000005640) was screened for any 9-mer peptides with some degree of homology to the Mtb target peptide. Homologous peptides biochemically similar to the cognate peptide, and/or having fewer than four site-wise amino acid differences (Hamming distance < 4) to the inhA_53-61_ peptide were identified. Further peptide details as described in *SI Appendix*, Table S1. See *SI Appendix* for details.

### Flow Cytometry of Peptide-Pulsed Cells.

K562-E*01:01 and K562-E*01:03 cell lines were pulsed with 10 µg/mL peptide for 2 h at 37 °C and 5% CO_2_. Peptide pulsed cells were washed once with buffer (PBS + 2% human AB serum + 2 nM EDTA) and stained for 30 min at 4 °C using anti-human HLA-E-PE (3D12; BioLegend) or anti-mouse IgG1κ-PE (MOPC-21; BD Pharmingen). Samples were washed twice and then analyzed using Sony SH800S (Sony Biotechnology). Cytometer files were analyzed with FlowJo software (FlowJo LLC).

### Soluble pHLA-E*01:01 and pHLA-E*01:03 Complexes.

Molecules were produced as previously described (49, 84, 85). For structural studies, nonbiotinylated soluble HLA-E peptide complexes were produced as described above but using HLA-E heavy chain without the AviTag™ sequence. The stability of all pHLA-E complexes was assessed by SPR using a BIAcore™ T200 instrument as previously described (49).

### Measurement of Molecules Binding Affinities and Kinetics.

Binding analysis of purified soluble TCRs, ImmTAB, and CD94/NKG2x receptors molecules to pHLA complexes was carried out by SPR, using either the BIAcore™ T200 (for weak affinity molecules) or 8K system (for affinity-enhanced molecules). See *SI Appendix* for details.

### IncuCyte Killing Assay.

In the IncuCyte S3 Live-Cell Analysis System (Essen Bioscience, Newark, UK), target cells were stained with CellTracker Deep Red Dye (Invitrogen, Carlsbad, CA). PBMC were added at a 10:1 ratio to targets with increasing concentrations of ImmTAB. In control wells, 1 nM of anti-CD3^mut^ ImmTAB-inhA or ImmTAB-inhA and 10 µM cognate peptide were used. Pan T and NK cells were added at a E:T ratio of 5:1 and 1:1, respectively. IncuCyte Caspase 3/7 Green Apoptosis Assay Reagent (Essen Bioscience) was added to all wells. Plates were incubated at 37 °C and 5% CO_2_ with images taken every 3 h. The number of apoptotic events/mm^2^ was calculated from two-color images.

### Mtb Infection and ToxiLight Assay.

PBMC from healthy blood donors (Ethical approval ref. 13/SC/0043) were isolated using density gradient centrifugation over Ficoll-Paque (GE Healthcare Life Sciences, UK). PBMC were cultured in T75 flasks either uninfected or infected with Mtb strain H37Rv at an MOI of 0.1. After 48 h at 37 °C and 5% CO_2_, PBMC were washed extensively and cultured at 1 × 10^6^ per well in 48-well flatbottom plates with or without 10 nM ImmTAB-inhA or CD3 nonbinding ImmTAB (anti-CD3^mut^ ImmTAB-inhA). After 24 h, supernatants were analyzed using the ToxiLight nondestructive cytotoxicity bioassay kit (Lonza, Switzerland) to detect adenylate kinase according to the manufacturer’s protocols.

### Infection of THP-1-Derived Macrophages with Mtb.

THP-1 cells were cultured in complete (FCS 10%, Glutamine 1%) RPMI medium plus gentamicin and penicillin at 37 °C and 5% CO_2_ in T75 flasks. After 3 d of culture, THP-1 were harvested, counted, and diluted at 1 × 10^6^/mL. A volume of 100 µL (1 × 10^5^ cells) of THP-1 cell suspension was added to wells of a 96-well plate and treated with PMA 100 IU/mL for 4 d. Then, cells were washed twice with PBS and were incubated with chemiluminescent Mtb strain H37Rv at a MOI 1:2 for 3 h in 50 mL 1640 RPMI medium without antibiotics. The wells were washed twice with PBS, filled with 200 mL complete RPMI medium without antibiotics, and incubated overnight at 37 °C and 5% CO_2_.

### Coculture of THP-1-Derived Macrophages and PBMC and CFU Assay.

Mtb-infected THP-1 macrophages were incubated with ImmTAB-inhA at different concentrations (1 nM, 2.5 nM and 5 nM) for 1 h in 50 μL complete 1640 RPMI medium without antibiotics. Then, cells were incubated with PBMC (2 × 10^5^ or 5 × 10^5^, or 1 × 10^6^, cell numbers) (*SI Appendix*, Table S5). After 24, 48, or 72 h incubation, target and effectors cells were lysed with 0.1% saponin and sonicated for 20 s. The number of colonies (CFUs) was counted as previously described (27, 86, 87).

## Supplementary Material

Appendix 01 (PDF)

Dataset S01 (XLSX)

Dataset S02 (XLSX)

Movie S1. **Antigen-dependent killing of target cells in the presence of ImmTAB-inhA**. Confocal imaging of PBMC (blue) co-cultured with a mixture of Ag+ (red; *inhA* transduced) and Ag– (yellow; non-transduced) A549 cells in the presence of 10 pM ImmTAB-inhA. Images were taken at 30-minute intervals over 3 days starting 7 h after addition of PBMC and displayed at 15 fps. Corresponding stills are shown in Figure 7B (top panels).

Movie S2. **No killing of target cells in the absence of ImmTAB-inhA**. Confocal imaging of PBMC (blue) co-cultured with a mixture of Ag+ (red; inhA transduced) and Ag– (yellow; non-transduced) A549 cells in the absence of ImmTAB-inhA. Images were taken at 30-minute intervals over 3 days starting 7 h after addition of PBMC and displayed at 15 fps. Corresponding stills shown in Figure 7B (bottom panels).

## Data Availability

All study data are included in the article and/or supporting information.
