# Supplementary material for: An HLA-E-targeted TCR bispecific molecule redirects T cell immunity against Mycobacterium tuberculosis
Source: Proc Natl Acad Sci U S A. 2024 May 1;121(19):e2318003121. doi: 10.1073/pnas.2318003121 (PMC11087797; doi:10.1073/pnas.2318003121)
Supplement: Supplementary file 1 — Appendix 01 (PDF) [file pnas.2318003121.sapp.pdf]

PNAS

Supporting Information for

An HLA-E-targeted TCR-bispecific redirects T cell immunity against *Mycobacterium tuberculosis*

Authors: Rachel L. Paterson[1,], Marco P. La Manna[2,3], Victoria Arena De Souza[1], Andrew Walker[1], Dawn Gibbs-Howe[1], Rakesh Kulkarni[1], Joannah R. Fergusson[1], Nitha Charles Mulakkal[1], Mauro Monteiro[1], Wilawan Bunjobpol[1], Marcin Dembek[1], Magdalena Martin-Urdiroz[1], Tressan Grant[1], Claire Barber[1], Diana J. Garay-Baquero[4], Liku Bekele Tezera[2], David Lowne[1], Camille Britton-Rivet[1], Robert Pengelly[1], Natalia Chepisiuk[1], Praveen K. Singh[1], Amanda P. Woon[1], Alex S. Powlesland[1], Michelle L. McCully[1], Nadia Caccamo[2,3], Mariolina Salio[1], Giusto Davide Badami[2,3], Lucy Dorrell[1], Andrew Knox[1], Ross Robinson[1], Paul Elkington[4], Francesco Dieli[2,3], Marco Lepore[1], Sarah Leonard[1], and Luis F. Godinho[1*]

*Luis F. Godinho

Email: luis.godinho@immunocore.com

This PDF file includes:

Materials and Methods
Figures S1 to S5
Tables S1 to S5
Legends for Datasets S1 and S2
Legends for Movies S1 to S2

Other supporting materials for this manuscript include the following:

Movies S1 to S2 (provided as MP4 video files)
Datasets S1 and S2 (separate files)

Cells

The Oxford A REC approved protocol 13/SC/0226 was used to obtain written consent for all blood donations and was fully approved by the National Research Ethics Committee South Central. Cell lines were purchased and grown as specified in Table S3. Cell line authentication and mycoplasma testing were routinely carried out by the LGC Standards Cell line Authentication Service and Mycoplasma Experience Ltd, respectively. Several of these cell lines were modified as specified in Table S3, and further described below. These cell lines cover a range of MHC Class I alleles and are therefore expected to express a variety of leader peptides naturally presented by HLA-E (Table S4). Generation of THP-1 β2m and CIITA knockout cells. In brief, sense and antisense sgRNAs were cloned into a Cas9D10A nickase plasmid using a similar method to that previously described (1). sgRNA sequences used to target β_2m were CTCGCGCTACTCTCTCTTTC (sense) and GGCCACGGAGCGAGACATCT (antisense); for targeting CIITA, sgRNA sequences were CTACCACTTCTATGACCAGA (sense) and CATCGCTGTTAAGAAGCTCC (antisense). The nickase plasmids containing targeting sgRNAs and Cas9 nickase linked with eGFP by T2A peptide were purified by NucleoBond Xtra Maxi Endotoxin-Free kit (MACHEREY-NAGEL, Bethlehem, PA, USA). THP-1 cells were transfected with the purified nickase plasmids using the Neon Transfection System (Thermo Fisher Scientific, Boston, MA, USA) according to the manufacturer's instructions. Cell populations with high eGFP expression were single cell sorted and β_2m/CIITA double knockout cell clones were validated by targeted DNA sequencing. Generation of HLA-E and inhA-expressing cell lines. In brief, human codon optimized sequence of single chain dimer β_2m-HLA-E*01:01, β_2m-HLA-E*01:03 and full length *inhA* (UniProtKB P9WGR1) were synthesized by GeneArt (Thermo Fisher Scientific) and cloned into the pELNS transfer vector using 5' NheI site and 3' SalI restriction sites. To generate lentivirus, the plasmids described above were transfected into HEK293T cells using Turbofect™ transfection reagent (Thermo Fisher Scientific). Lentiviral particles were harvested and used to transduce cell lines as indicated in Table S3. Throughout the text, THP-1 β_2m and CIITA knockout cells lentivirally transduced with β_2m-HLA-E*01:01 or β_2m-HLA-E*01:03 single chain dimer are abbreviated to THP-1-E*01:01 or THP-1-E*01:03, respectively, and THP-1-E collectively. Similarly, K562 cells lentivirally transduced with β_2m-HLA-E*01:01 single chain dimer or β_2m-HLA-E*01:03 single chain dimer are abbreviated to K562-E*01:01 or K562-E*01:03, respectively.

Mtb gene expression analysis

To evaluate expression of Mtb genes, data from Homolka et al. (2) were analyzed through NCBI's Gene Expression Omnibus (accession GSE21114). Intracellular expression data were extracted, resulting in expression values for 17 distinct isolates with biological and technical replicates. In each microarray experiment, the average of replicates was taken before performing a simple per-

gene ranking of expression data from each experiment. This analysis allows identification of Mtb genes which are constitutively expressed across experiments.

HLA-E genotyping

DNA was extracted from 0.5 – 3 × 10^6 cells using QIAprep Spin mini kit (Qiagen). Genomic DNA was amplified using forward primer 5'-GGTCTCACACCCTGCAGTGGA-3' and reverse primer 5'-AGCCCTGTGGACCCTCTT-3'. DNA was PCR amplified with Phusion High Fidelity DNA polymerase (New England Biolabs) and migrated in 1.5 % agarose gel. A band of ~280 bp was excised and purified using Nucleospin Gel and PCR Clean-up kit (MACHEREY-NAGEL). Sanger sequencing was used to determine the polymorphism in codon 107 of HLA-E.

Mtb peptides and human peptides homologous to Mtb target

To identify peptides that are potential HLA-E binders, netMHCpan4.0 peptide-HLA binding prediction algorithm uses data from eluted mass spectrometry peptides and experimental MHC-binding data to train neural networks to quantitatively predict per-HLA-type peptide binding propensities. For the identification of human peptides homologous to the Mtb target for cross-reactivity screening, biochemical similarity is defined by a "BLOSUM score", which was calculated as the mean of the position-wise BLOSUM62 substitution probability values between two peptides. Peptides that differed by 4 or less amino acids and were biochemically similar to inhA$_{53-61}$ were identified (n=141)(3). These peptides were then screened and ranked for predicted binding to HLA-E*01:01 using netMHCpan4.0 using a conservative binding threshold of <5%. The Genotype-Tissue Expression portal (GTEx; https://www.gtexportal.org/home) was then used to analyze expression of genes containing the human peptides with some similarity to the target. Nine peptides were selected for screening (M1-M9; Table 1) due to their high BLOSUM score, high predicted binding and a median transcript per million (TPM) in high-risk tissues. Peptides were obtained by chemical synthesis (Peptide Protein Research Ltd, Fareham, UK) and solubilized in DMSO to a concentration of 4 mg/mL prior to use.

Immunopurification and quantification of inhA$_{53-61}$ peptide presented on HLA-E complexes

HLA-A*02:01/β$_2$m (A2B2M) and inhA were ectopically expressed in THP1 and U937 cells using lentiviral transduction. Cells were cultured according to suppliers' instruction, harvested, and stored at – 80°C prior to analysis. HLA complexes were purified by immunoaffinity using sequential HLA-E-specific (3D12) and HLA-A*02-specific (BB7.2) antibodies. Data was acquired on an Orbitrap Fusion Tribrid Mass Spectrometer (ThermoFisher) and analyzed using Thermo Freestyle software. Briefly, cells were lysed in buffer containing non-ionic detergent NP-40, cell debris was removed by centrifugation and supernatant passed over resins containing HLA-A*02-specific and HLA-E-specific antibodies immobilized on a protein A- or protein G-Sepharose respectively. Columns were

washed and complexes eluted in 0.5% triflouroacetic acid (TFA). Immunopurified material was desalted and reduced in volume by vacuum centrifugation prior to reconstitution in 0.1% TFA, 5% acetonitrile and analysis by liquid chromatography-parallel reaction monitoring-mass spectrometry (LC-PRM-MS). Peptides were loaded onto an Acclaim PepMap 100 trap column (100 µm x 20 mm, ThermoFisher) and separated using an Easyspray column (75µm x 500mm, ThermoFisher). Data was acquired on an Orbitrap Fusion Tribrid Mass Spectrometer (ThermoFisher) using the following settings. A full MS1 scan was recorded at 120K resolution (AGC 3E5, 50ms) after quadrupole isolation (200 – 1200 m/z range). Precursor ions of target peptides were selected for MS2 by tMS (targeted MS). Quadrupole isolation was set to 1.2 Da, HCD fragmentation to 28 NCE and MS2 spectra recorded in the Orbitrap at 60K resolution (AGC 1E6, 120 ms). Start/End times were included in the method with a 15-minute window placed around the expected peptide elution time. Stable-isotope labelled (SIL) peptides (JPT technologies) were introduced into each sample at an exact molar amount of 100 femtomoles, immediately prior to analysis. Data was analyzed using Thermo Freestyle software. For quantitative estimates of target peptide, the LC area of 3 fragment ions from native and SIL peptide species were extracted with a 10 ppm mass tolerance. Peak integration was enabled using the following settings: baseline window 150, area noise factor 1, peak noise factor 1. The molar amount of the native peptide was calculated for each fragment ion using the area ratio between the SIL and native peptide. The molar amount of 3 fragment ions was averaged and copy numbers were calculated after accounting for the number of cells.

Generation of soluble TCRs, ImmTAB and CD94/NKG2x molecules

Soluble TCRs and ImmTAB molecules were produced as previously described (4-6). Briefly, enhanced affinity TCRs were generated from a wild-type TCR specific for the Mtb antigen $inhA_{53-61}$ (RLPAKAPLL) bound to HLA-E using directed molecular evolution and phage display selection (7). The human ectodomains of NKG2A (residues 94–233) and NKG2C (residues 94–231) both with N-terminal Fc knob (T366W) tag, and CD94 (residues 34–179) with N-terminal Fc hole (T366S, L368A and Y407V) tag were cloned into pCDNA3.1 vectors (containing IL-2 signal sequence). Protein constructs were co-transfected into ExpiCHO cells and cultured according to ExpiCHO™ Expression System Kit manufacturer specifications (Thermo Fisher Scientific). Cell culture supernatants were loaded into protein-L HiTrap column (GE Healthcare Life Sciences) and further purified using Superdex 200 HR10/300 column (GE Healthcare Life Sciences). The purity of heterodimers was determined by SDS-PAGE analysis.

Measurement of molecules binding affinities and kinetics

Binding analysis of purified soluble test TCRs, ImmTAB and CD94/NKG2x receptors molecules to pHLA complexes was carried out by SPR. Briefly, biotinylated cognate pHLAs were immobilized onto a streptavidin-coupled CM5 sensor chip. Flow cell one was loaded with free biotin alone to act

as a control surface. All measurements were performed at 25°C in Dulbecco's PBS buffer (Sigma-Aldrich, St Louis, MO, USA) containing P20 surfactant (0.005%) at a flow rate of 10 – 30 µL/min for the T200 or 50 – 60 µL/min for the 8K. Binding profiles were determined using either steady state affinity analysis (for weak affinity molecules) or single cycle kinetic analysis (for affinity-enhanced molecules). For steady state affinity analysis, weak affinity molecules were injected at top concentrations ranging between 0.4 – 50 µM preceded by seven or eleven injections using serial 2-fold dilutions. For single cycle kinetics, affinity-enhanced molecules were injected at top concentrations ranging between 100 – 1000 nM preceded by four injections using serial 2-fold dilutions. K_D values were calculated assuming Langmuir binding and data was analyzed using a 1:1 binding model (GraphPad Prism v8.3.0 for steady state affinity analysis and Biacore Insight Evaluation v2.0.15.12933 for single cycle kinetics analysis).

Protein crystallization, x-ray data collection and structure determination

The TCR-pMHC complexes were prepared by mixing purified TCR and untagged pMHC at molar ratios of 1:1.15 and concentrating to approximately 10 mg/mL. The crystallization trials were set up by dispensing 150 nL of protein solution plus 150 nL of reservoir solution in sitting-drop vapor diffusion format in two-well MRC Crystallization plates using a Gryphon robot (Art Robbins). The plates were maintained at 20°C in a Rock Imager 1000 (Formulatrix) storage system. Diffraction quality crystals of a42b20 TCR-HLA-E-inhA were obtained in 0.2 M calcium acetate, 0.1 M imidazole pH 8.0, 10% (w/v) PEG 8000. X-ray diffraction data were collected at the Diamond Light Source (Oxfordshire, UK) beamline I04. Diffraction images were processed using dials (8) and dials.scale (9) through the xia2 (10) automated data-processing suite. Structures were solved by molecular replacement using PDB 6GH1 (chains A and B) (11) and 4WW2 (chains A and B) (12) as a search model for HLA-E and TCR, respectively, in Phaser (13). Models were built using iterative cycles of manual model building in COOT (14) and refinement using Refmac5 (15) in the CCP4 suite (16). Additional model validation was performed using PDB_REDO (17, 18). The data processing and refinement statistics are listed in Table S2. The structural figures were prepared using PyMOL (Schrödinger).

Enzyme-linked immunospot (ELISpot) assays

IFN-γ ELISpot assays were performed according to the manufacturer's recommendations (BD Biosciences). Briefly, target cells were plated in triplicate at 5×10^4 cells per well and incubated with healthy donor PBMC at 5×10^4 cells per well. For peptide-pulsing experiments, target cells were incubated with various concentrations of peptide (Peptide Protein Research Ltd) for 2 h and washed twice before plating with ImmTAB-inhA molecules and PBMC. Plates were incubated overnight at 37°C/5% CO_2 followed by IFN-γ detection, and spots were quantified using the BD

ELISpot reader (Immunospot Series 5 Analyzer, Cellular Technology Ltd, Shaker Heights, OH, USA). See SI Appendix for details on antibodies used for blocking assays.

Western blotting

Cells were lysed in RIPA buffer (Sigma-Aldrich) for 30 min on ice and centrifuged at 17,000 xg for 10 min at 4°C. Pellet was discarded, and the protein levels in the supernatant were estimated with BCA protein assay kit (Pierce Biotechnology, Waltham, MA, USA). A sample of 20 µg was separated by SDS-PAGE (Invitrogen) and transferred onto 0.45 mM nitrocellulose membrane (Invitrogen). See SI Appendix for details on antibodies used.

Antibodies for blocking assays and western blotting

For ELISpot assays including monoclonal antibodies, endotoxin- and azide-free unconjugated monoclonal antibodies were generated to order (InVivo BioTech Services, Germany) and added at 10 µg/mL for the duration of the assay. Antibodies used were anti-HLA-E (3D12), anti-HLA-A2 (BB7.2) and anti-HLA-B7 (BB7.1). For western blotting, primary antibodies were used to probe the membrane: mouse anti-HLA-E (MEM-E/07; Abcam, Cambridge, UK); rabbit anti-HLA-A (EP1395Y; Abcam); mouse anti-β2m (D8P1H; Cell Signaling Technology Inc., Danvers, MA, USA) and rabbit anti-b-tubulin (EPR16778; Abcam). Detection was carried out using HRP-conjugated goat anti-mouse IgG or donkey anti-rabbit IgG (Abcam), followed by the addition of ECLWestern Blotting Substrates (Pierce Biotechnology). Protein bands were visualized with ChemiDoc XRS Imaging System (Bio-Rad, Watford, UK).

Degranulation and proliferation assay

Degranulation in T cell subsets was assessed by adding CD107a-AlexaFluor647 (Biolegend) at the start of co-culture and analyzed after 48 h incubation at 37°C/5% CO2. Proliferation was assessed with Cell Trace Violet (Invitrogen) and analyzed after 5 days of co-culture. T cell subsets were gated from live lymphocytes using Zombie Live Dead dye (BioLegend) and defined as γδ T cells ($CD3^+TCR\gamma\delta^+$), $CD4^+$ T cells ($CD3^+CD4^+CD8^-TCR\gamma\delta^-$), $CD8^+$ T cells ($CD3^+CD4^-CD8^+TCR\gamma\delta^-$), and MAIT cells ($CD3^+TCR\gamma\delta^-CD161^{++}V\alpha7.2^+$), by CD3 Pacific Blue (BioLegend) or AlexaFluor 700 (BD Biosciences), CD4 PerCPCy5.5 (BioLegend), CD8 FITC (BD Biosciences), pan TCRγδ-PE (BioLegend) or TCR Vδ2 Brilliant Violet 711 (BioLegend), CD161 PE (Miltenyi Biotec) or APC (BioLegend), and TCR Vα7.2 PECy7 (BioLegend). Data was acquired on a Sony MA900 (Sony Biotechnology) and analyzed by FlowJo (FlowJo LLC).

Cytokine analysis

Cytokine, chemokine, and growth factor release was measured with the Human Cytokine Magnetic 30-Plex Panel (Thermo Fisher Scientific) for the Luminex platform, in accordance with the manufacturer's instructions. Analyte levels (pg/mL) were determined with the Luminex MAGPIX System (Merck Millipore, Burlington, MA, USA) using xPONENT software or the Bio-Plex 200 System from BioRad using the Bio-plex manager software. Data were further analyzed using R software.

Opera Phenix™ killing assay

ImmTAB-mediated target cell apoptosis was imaged using the Opera Phenix High Content Screening System and analyzed with the Harmony high-content imaging and analysis software (PerkinElmer, Waltham, MA, USA). For assays with mixed target cell populations, inhA antigen transduced (Ag+) A549 cells stained with CellTracker Deep Red (Invitrogen) and antigen negative (Ag-) A549 cells stained with CellTracker Red CMTPX (Invitrogen) were mixed 1:1 and 6,000 targets per well were plated in a flat-bottomed Phenix CellCarrier Ultra microwell plate (PerkinElmer). Targets were cultured overnight at 37°C/5% CO_2 before addition of 10 pM ImmTAB-inhA and an E:T of 10:1 with PBMC effectors stained with Cell Tracker Violet (Invitrogen). Apoptosis was tracked by addition of Caspase 3/7 Green Apoptosis Assay Reagent (Essen Bioscience). Images were taken at 30-minute intervals over 3 days starting 7 h after addition of PBMC and displayed at 15 fps.

H37Rv culture and infection

Mtb H37Rv were routinely cultured in Middlebrook 7H9 medium (BD Biosciences, Oxford) supplemented with 10% ADC enrichment (SLS), 0.2% glycerol and 0.02% Tween 80. For all experiments, bacterial cultures were grown to optical density 600 nm (OD_{600}) of 0.6 (approx. 1x 10^8 CFU/mL). Host cells were then infected with mycobacteria at an MOI of 0.1.

RNA extraction from H37Rv liquid culture and PBMC co-cultures

For bacteria grown in 7H9 broth (OD_{600} of 0.25) and in co-culture with PBMC, total RNA was extracted as reported previously (19). RNA was further purified with the Qiagen Rneasy mini kit (Qiagen), subjected with on-column Dnase digestion with the Rnase-free Dnase set (79254; Qiagen), repurified with a RNeasy mini kit, and eluted in 50 µl of Rnase- and Dnase-free water (Fisher Scientific) until used.

qRT-PCR

Total RNA was isolated from n≥3 biological replicates (individual PBMC donors or bacterial lots) using the RNeasy mini kit (Qiagen) and reverse-transcribed into cDNA with the iScript™ Advanced

cDNA Synthesis Kit for RT-qPCR (Bio-Rad) according to the manufacturer's instructions. *inhA* expression levels were quantified by qRT-PCR using the QuantiFAST SYBR Green PCR Kit (Qiagen) on the Quantstudio 6 real-time PCR system (Life Technologies). See SI Appendix for details. Primers were custom designed to detect either the native form of the *inhA* gene (forward 5'-TGGACGGCAAACGGATTCT-3'; reverse 5'-CCTGCTCCTGGGCTACC-3'), or the codon optimized *inhA* ORF expression plasmid used for lentiviral transductions of cell lines (forward 5'-CAAGGGCATTCACATCAGCG-3'; reverse 5'-CATCCAGTTGTAGGCAGGCA-3'). Reactions were run in duplicates with 250 nM primers under the following conditions: 95◦C for 5 min, 40 cycles of 95◦C for 10 s and 60◦C for 30 s, followed by a melt curve. *inhA* plasmids with a known transcript copy number were analysed as a 7-point serial dilution standard curve for measurement of PCR efficiency and transcript number quantification. Results were then normalized to 100 ng RNA input for comparison to the bacterial culture. Three house-keeping genes (MRPL18, RNF20 and ZC3H15) were used to control for quality of human cell samples by calculating the geometric mean between the genes. The Normalized Relative Quantity (NRQ) of *inhA* was calculated using the efficiency-adjusted Delta-Delta Ct method.

<u>Subjects enrolled in the study</u>

We tested 33 healthy donors and 8 IGRA positive donors (Table S7) enrolled by clinicians, respectively at the Division of Infectious Diseases University Hospital, Palermo, Italy. Each participant provided written informed consent, and the research conducted according to ethical standards. We considered donors having had Mtb exposure the subjects positive for the IGRA test without clinical or microbiological, biomolecular or microscopy findings. Peripheral blood was drained in EDTA tubes by venipuncture. PBMC were obtained by gradient separation using FICOLL. PBMC were counted with Turk and diluted at a concentration of $2x10^6$/mL and used for experiments.

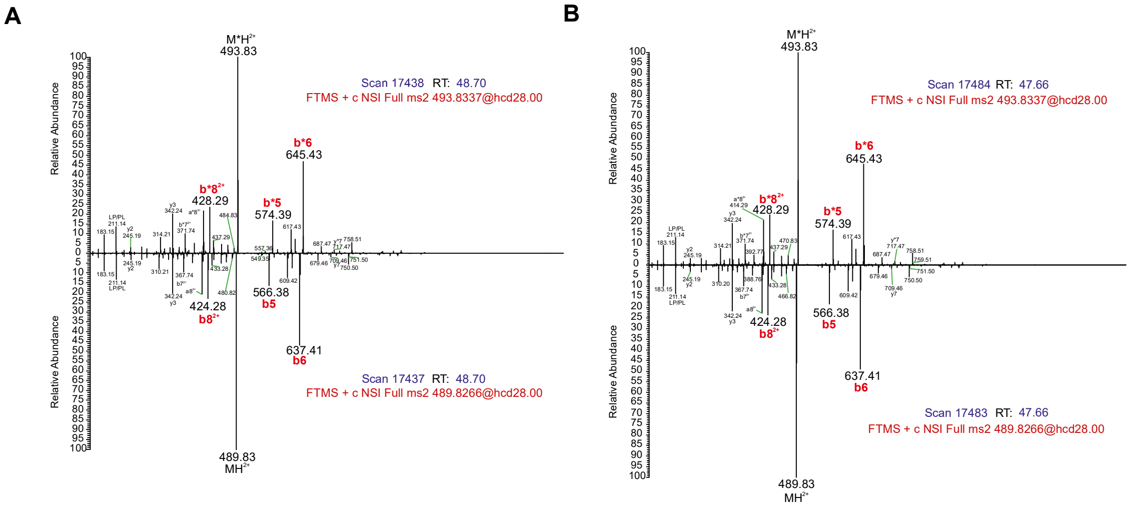

Fig. S1. Assigned MS/MS spectra for inhA$_{53\text{-}61}$ identified in immunopurified HLA-E complexes extracted from THP1 (A) and U937 (B) cells. Spectra are presented as mirrored images with SIL peptide above and native peptide below. Major ions are labelled, as are dominant fragment ions. Ions used for quantitation are highlighted in red.

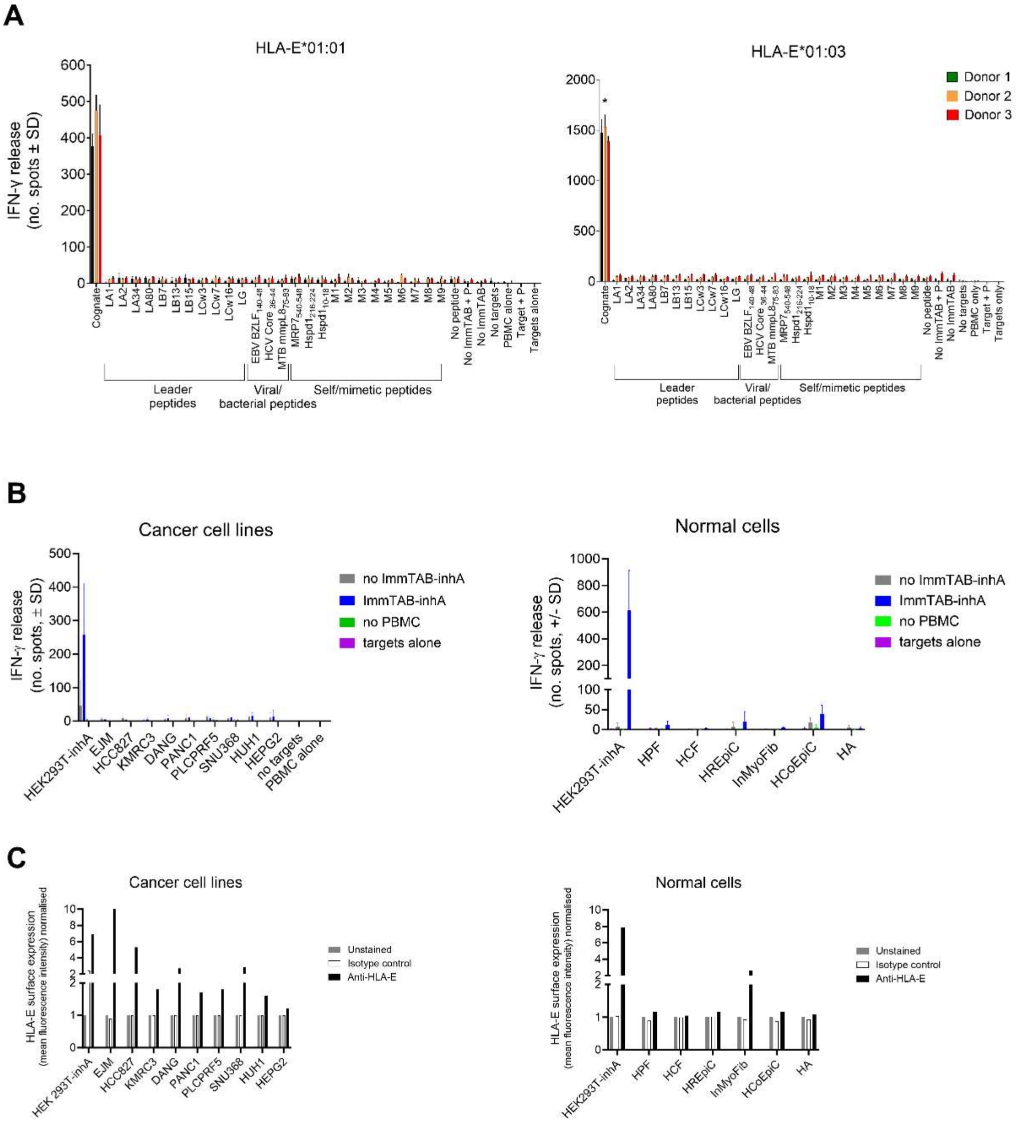

Fig. S2. Specificity of ImmTAB-inhA for the cognate peptide presented by HLA-E

(A) IFN-γ responses from 3 healthy donor PBMC in the presence of 1 nM ImmTAB-inhA and THP-1-E*01:01 cells (left panel) and THP-1-E*01:03 cells (right panel) pulsed with 10 µg/mL of either cognate $inhA_{53-61}$ peptide or other indicated peptides. Controls included no peptide, no ImmTAB-inhA, no targets, PBMC only or targets +/- cognate peptide (P). **(B)** IFN-γ responses from 3 healthy donor PBMC were measured in the presence of ImmTAB-inhA and a panel of cancer cell lines (left panel) or normal cells (right panel) expressing different HLA-alleles as detailed in SI Appendix, Table S4. Controls included no ImmTAB-inhA, no PBMC, no target cells, and PBMC or target cells only. **(C)** The same panel of cancer cell lines (left) and normal cells (right) was analyzed for HLA-E surface expression by flow cytometry. Values are normalized to unstained cells.

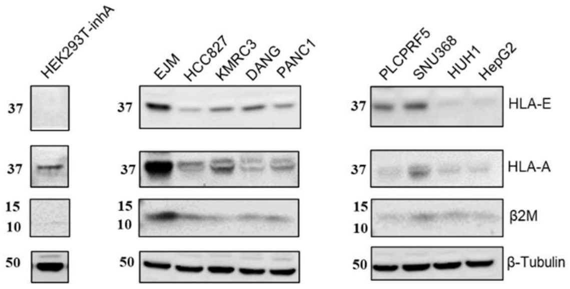

Fig. S3. Western blot assessment of HLA expression. Expression of HLA-E, HLA-A, beta-2-microglobulin (β2m) in lysates of the indicated cell lines were assessed by Western blot. β-tubulin was included as a loading control.

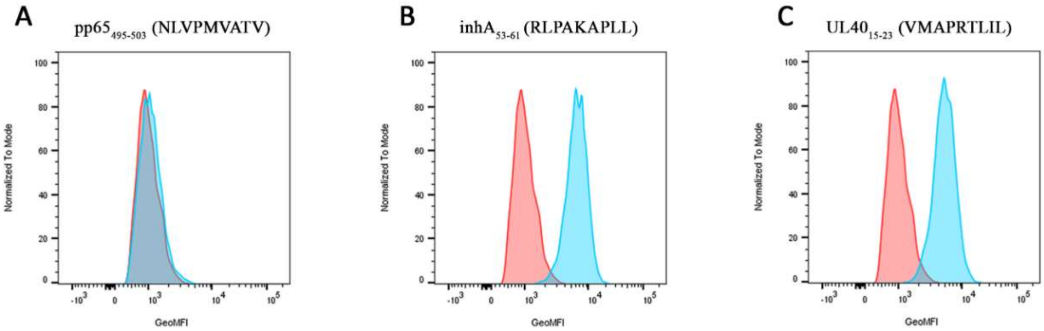

Fig. S4. Stabilization of HLA-E surface expression by inhA$_{53\text{-}61}$ peptide. The murine TAP2-deficient T cell lymphoma RMA-S cell line co-transfected with human β_2-microglobulin and HLA-E*01:03 allele (RMA-S/HLA-E, kindly provided by J.E. Coligan, Laboratory of Immunogenetics, NIAID, Rockville, MD) was used in the experiments. RMA-S/HLA-E cells were re-suspended in complete Roswell Park Memorial Institute medium at 1×10^6 cells/mL and incubated either alone or in the presence of the CMV-derived peptide pp65$_{495\text{-}503}$ (NLVPMVATV) used as negative control, inhA$_{53\text{-}61}$ or CMV-derived peptide UL40$_{15\text{-}23}$ (VMAPRTLIL), all at 10 µg/mL final concentration. After an overnight incubation at 37°C the cells were washed with PBS to remove free peptides. Next, HLA-E surface expression was monitored staining with the PE-conjugated anti-human-HLA-E, clone 3D12, Miltenyi Biotec GE. The overlays represent the comparison of fluorescence intensity of RMA-S cells unpulsed (red) and pulsed with negative control peptide (light blue, A), UL40 peptide (light blue, B) and inhA$_{53\text{-}61}$ peptide (light blue, C). GeoMFI values were as follows: unpulsed RMA-S = 1007; RMA-S pulsed with negative control peptide, UL40 peptide and inhA$_{53\text{-}61}$ peptide = 1089, 6129, and 4913, respectively.

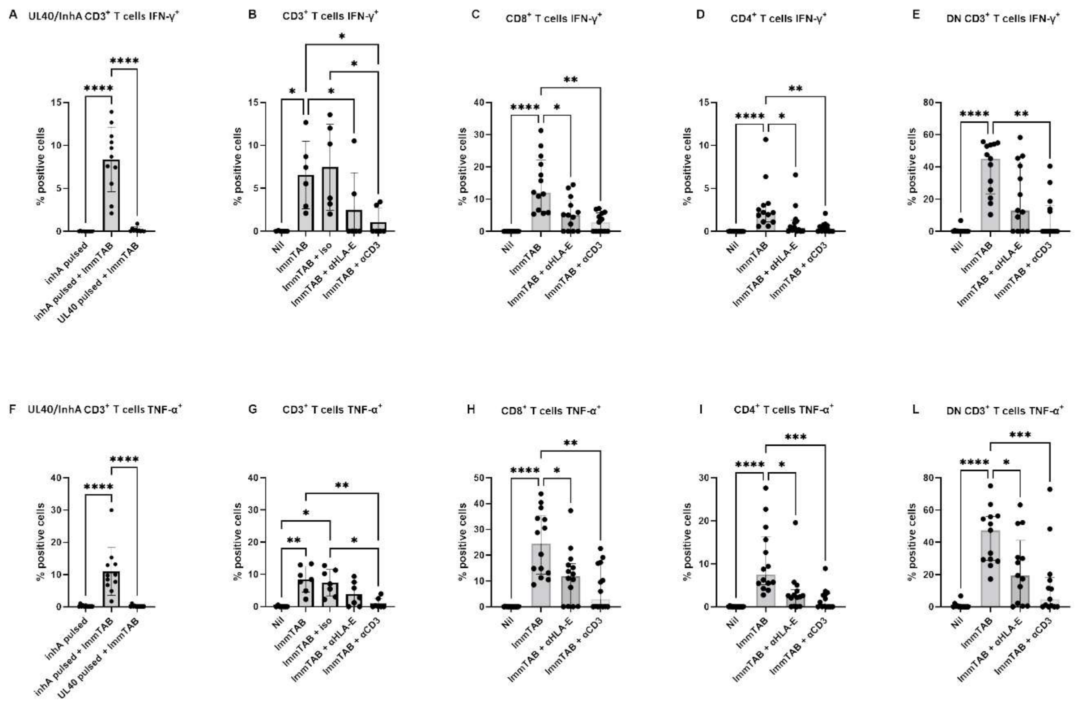

Fig. S5. ImmTAB-inhA redirects $CD3^+$ T cells to RMA-S/HLA-E cells pulsed with the cognate peptide. TAP2-deficient T-cell lymphoma RMA-S-cell line transfected with human β2-microglobulin and HLA-E*01:03 allele (RMA-S/HLA-E, were kindly provided by J. E. Coligan, Laboratory of Immunogenetics, NIAID, Rockville, MD, USA). RMA-S/HLA-E cells were resuspended in complete RPMI medium at 2×10^6 cells/mL and incubated (37°C 5% CO_2) alone or pulsed for 1 h with $inhA_{53-61}$ or CMV-derived UL40 peptide (as positive control) at the concentration of 10 µg/mL. After incubation, cells were washed with PBS to remove free peptides. Peptide-pulsed RMA-S/HLA-E cells were incubated with PBMC at a *ratio* of 1:10 in the presence of monensin, for 4 h at 37°C in 5% CO_2, in the presence of anti-HLA-E mAb (eBioscience™, clone 3D12 HLA-E, Thermo Fisher, MA, USA), anti-CD3 mAb (Leica Biosystems, clone LN10, Wetzlar, Germany) or isotype control mAb, all used at the final concentration of 10 µg/mL/10^6 cells. After incubation, the cells were harvested, washed and stained with live/dead marker (Viobility™ Fixable Dyes, Miltenyi Biotec, Germany) for 15 minutes at room temperature, then with mAbs to human CD3 PerCP-Vio® 700 (clone REA613, Miltenyi Biotec, Germany), human CD4 PE-Vio® 770 (clone REA623, Miltenyi Biotec, Germany) and human CD8 APC (clone REA734, Miltenyi Biotec, Germany) for 20 minutes at room temperature. After surface staining, cells were fixed/permeabilised (eBioscience c.n. 88-8824-00 Thermo Fisher Scientific Waltham, Ma, USA.) and stained at room temperature for 30 min with anti-human IFN-γ FITC (clone REA600, Miltenyi Biotec, Germany) and anti-human TNF-α PE (clone REA656, Miltenyi Biotec, Germany) or isotype-matched control mAbs. Samples were acquired on a FACSLyric™ flow cytometer (BD Bioscience San Jose CA, USA) and analysed with

FlowJo v10 (BD Bioscience San Jose, CA, USA): a minimum of 100 000 events was acquired for each sample. Initial gating was performed on single cells, followed by the selection of live cells and FSC/SSC to gate on lymphocytes. T cells were gated as $CD3^+$ cells and further selected for $CD8^+$ and $CD4^+$ or double negative (DN) to measure cytokine secretion. The graphs represent the median ± IQR of each experimental point, and the Kruskal-Wallis test assessed any statistically significant difference. *= $p \leq 0.05$, **= $p \leq 0.01$, ***= $p \leq 0.001$, ****= $p \leq 0.0001$.

Table S1. Details of peptides used for ELISpot and/or peptide pulsing assays. *Human 9mer peptides (M1-M9) identified from the human proteome with similar properties to the target peptide, as described in Materials and Methods.

**Peptide name**	**Abbreviated peptide name**	**Description**	**Peptide Sequence**
$inhA_{53\text{-}61}$	$inhA_{53\text{-}61}$	Mtb target peptide (20)	RLPAKAPLL
Leader A1	LA1	HLA leader sequence peptides (21)	VMAPRTLLL
Leader A2	LA2		VMAPRTLVL
Leader A34	LA34		IMAPRTLVL
Leader A80	LA80		VMPPRTLLL
Leader B7	LB7		VMAPRTVLL
Leader B13	LB13		VTAPRTLLL
Leader B15	LB15		VTAPRTVLL
Leader Cw3	LCw3		VMAPRTLIL
Leader Cw7	LCw7		VMAPRALLL
Leader Cw16	LCw16		VMAPQALLL
Leader G	LG		VMAPRTLFL
EBV $BZLF1_{40\text{-}48}$	EBV $BZLF1_{40\text{-}48}$	Epstein-Barr virus peptide (22)	SQAPLPCVL
HCV $Core_{36\text{-}44}$	HCV $Core_{36\text{-}44}$	Hepatitis C virus peptide(23)	LLPRRGPRL
MTB $MmmpL8_{75\text{-}83}$	MTB $mmpL8_{75\text{-}83}$	Mtb peptides (20)	ILPSDAPVL
$Rv1518_{240\text{-}248}$	$Rv1518_{240\text{-}248}$		VMATRRNVL
$Rv2997_{470\text{-}478}$	$Rv2997_{470\text{-}478}$		RMPPLGHEL
$MRP7_{540\text{-}548}$	$MRP7_{540\text{-}548}$	Self-peptides (24, 25)	ALALVRMLI
$Hspd1_{216\text{-}224}$	$Hspd1_{216\text{-}224}$		GMKFDRGYI
$Hspd1_{10\text{-}18}$	$Hspd1_{10\text{-}18}$		QMRPVSRVL
Mimetic 1	M1	Potential mimetic peptides from the human proteome*	RLPASAPLF
Mimetic 2	M2		RLPARTPLF
Mimetic 3	M3		QVPAKAPLA
Mimetic 4	M4		RLPGEAPVI
Mimetic 5	M5		SLPAKADLL
Mimetic 6	M6		KLPANHPLL
Mimetic 7	M7		RKPAMSPLL
Mimetic 8	M8		RLPLKLPTL
Mimetic 9	M9		RLPAWQPIL

Table S2. Data processing and refinement statistics for a42b20-HLA-inhA TCR-pMHC complex. Values in parentheses refer to the outer resolution shell.

**Molecule**	**a42b20-HLA-E_{inhA}**
**Data collection**	
PDB code	8QFY
Space group	C 1 2 1
Unit cell dimensions	a=326.83, b=79.88, c=104.07<br>α=90.00°, β=96.89°, γ=90.00°
X-ray source	DLS I04
Wavelength (Å)	0.97950
Resolution range (Å)	2.33-82.90 (2.33-2.37)
Completeness (%)	100 (100)
Multiplicity	6.8 (6.0)
I/σ I	11.3 (0.3)
R_{merge}	0.121 (3.203)
R_{pim}	0.050 (1.430)
$CC_{1/2}$	0.987 (0.336)
Unique reflections	114168 (5689)
**Refinement**	
Rwork / Rfree (%)	24.25 / 28.86
RMS (bonds)	0.006
RMS (angles)	1.406
Mean B-factor ($Å^2$)	74.98

Table S3. Cell lines, suppliers and culture conditions. R10 and R20 = RPMI media supplemented with 1% (v/v) penicillin/streptomycin, 2 mM L-glutamine, and 10% or 20% fetal calf serum (FCS) respectively; D10 and E10 are DMEM and EMEM supplemented as R10. *ATCC = American Type Culture Collection (Manassas, USA), DSMZ = German Collection of Microorganisms and Cell Cultures (Braunschweig, Germany), ECACC = European Collection of Authenticated Cell Cultures (Salisbury, UK), IZSBS = Istituto Zooprofilattico Sperimentale della Lombardia e dell'Emilia Romagna (Brescia, Italy), JCRB = Japanese Collection of Research Bioresources Cell Bank (Osaka, Japan)

**Cell line**	**Supplier***	**Catalogue number**	**Media**	**Modifications**
A549	ATCC	CCL-185	F-12K + 10% FCS	Lentiviral transduction with inhA
CALU3	ATCC	HTB-55	E10	
DANG	DSMZ	249	R10	
EJM	DSMZ	ACC560	IMDM + 20% FCS	
HCC827	DSMZ	ACC 566	R20	
HEK 293T	DSMZ	ACC635	D10	Lentiviral transduction with inhA
HEPG2	ATCC	HB 8065	E10	
HUH1	JCRB	JCRB0199	D10	
K562	ATCC	CCL-243	R10	Lentiviral transduction with β_2m-HLA-E*01:01 or β_2m-HLA-E*01:03 single chain gene-fusion constructs (K562-E*01:01 and K562-E*01:03 respectively)
KMRC3	JCRB	JCRB1012	D10	
PANC1	IZSBS	BSTCL 49	D10	
PLC/PRF/5	Public Health England	85061113	D10	
SNU368	Korean Cell Line Bank	368	R10	
THP-1	ATCC	TIB202	R10	CRISPR-Cas9 knockout of β_2m and CIITA; lentiviral transduction with β_2m-HLA-E*01:01 or β_2m-HLA-E*01:03 single chain gene-fusion constructs; lentiviral transduction with inhA
U937	ECACC	8501 1440	R10	Lentiviral transduction with inhA
HCOEpiC	ScienCell	ScienCell-9763	Colonic Epithelial Cell Medium kit	
InMyoFib	Lonza	Lonza-0000362793	Smooth Muscle Cell Growth Medium-2 BulletKit	
HCF	Lonza	Lonza-CC-2904-0000662122	Fibroblast growth medium-3 kit	

HPF	Promocell	Promocell-C-12360-433Z024	Fibroblast Growth Medium-2 BulletKit	
HA	ScienCell	ScienCell -1800-3911	Astrocyte Medium kit	
HREPiC	Promocell	Promocell-C-12665-414Z005	Renal Epithelial Cell Growth Medium 2 kit	

Table S4. MHC Class I alleles and leader peptides of cell lines. Leader peptide sequences as described in Table 1. HLA-E genotyping was performed in house as described in materials and methods. ND – not determined.

Cell line	HLA-A alleles	HLA-B alleles	HLA-C alleles	HLA-E alleles	Equivalent HLA leader peptides
A549	A*02:06<br>A*24:02	B*15:01<br>B*46:01	C*01:02<br>C*04:01	E*01:01<br>E*01:03	A2, B15, Cw3
DANG	A*02:01<br>A*02:01	B*07:02<br>B*13:02	C*06:02<br>C*07:02	E*01:03<br>E*01:03	A2, B13, B15, Cw3, Cw7
EJM	A*01:01<br>A*32:01	B*08:01<br>B*55:01	C*03:03<br>C*07:01	E*01:01<br>E*01:03	A1, B7, B13, Cw3, Cw7
HCC827	A*11:01<br>A*11:01	B*35:01<br>B*52:01	C*12:02<br>C*12:02	E*01:03<br>E*01:03	A1, B15, Cw3
HEK 293T	A*02:01<br>A*02:01	B*07:02<br>B*07:02	C*07:01<br>C*07:01	E*01:03<br>E*01:03	A2, B7, Cw7
HEPG2	A*02:01<br>A*24:02	B*35:14<br>B*51:08	C*04:01<br>C*16:02	E*01:01<br>E*01:01	A2, B15, Cw3, Cw16
HUH1	A*02:06<br>A*11:01	B*15:01<br>B*38:02	C*01:02<br>C*07:02	E*01:01<br>E*01:03	A1, A2, B15, Cw3, Cw7
K562	Null	Null	Null	Null	N/A
KMRC3	A*11:01<br>A*24:02	B*51:01<br>B*55:02	C*03:03<br>C*14:02	E*01:03<br>E*01:03	A1, A2, B13, B15, Cw3, Cw7
PANC1	A*02:01<br>A*11:01	B*38:01<br>B*38:01	C*12:03<br>C*12:03	E*01:01<br>E*01:03	A1, A2, B7, Cw3
PLCPRF5	A*03:01<br>A*33:01	B*42:02<br>B*53:01	C*04:01<br>C*17:01	E*01:03<br>E*01:03	A1, B7, B15, Cw3, Cw7
SNU368	A*02:06<br>A*24:02	B*15:01<br>B*46:01	C*01:02<br>C*04:01	E*01:03<br>E*01:03	A2, B15, Cw3
THP-1	A*02:01<br>A*02:01	B*15:11<br>B*15:11	C*03:03<br>C*03:03	E*01:03<br>E*01:03	A2, B15, Cw3
U937	A*03:01<br>A*31:01	B*18:01<br>B*51:01	C*01:02<br>C*07:01	E*01:01<br>E*01:03	A1, B13, B15, Cw3, Cw7
HCOEpiC	A*03:01<br>A*11:01	B*07:02<br>B*18:01	C*07:01<br>C*07:02	ND	A1, B7, B13, Cw7
InMyoFib	A*02:01<br>A*03:01	B*07:02<br>B*27:03	C*02:02<br>C*07:02	ND	A1, A2, B7, B13, Cw2, Cw7
HCF	A*24:02<br>A*24:02	B*15:01<br>B*56:01	C*01:02<br>C*03:03	ND	A2, B13, B15, Cw3
HPF	A*02:01<br>A*24:02	B*07:02<br>B*35:01	C*04:01<br>C*07:02	ND	A2, B7, B15, Cw3, Cw7
HA	A*11:01<br>A*30:01	B*42:01<br>B*50:01	C*06:02<br>C*17:01	ND	A1, B7, B15, Cw3, Cw7
HREPiC	A*11:01<br>A*24:02	B*38:02<br>B*51:01	C*07:01<br>C*07:04	ND	A1, A2, B7, B15, Cw7

Table S5. Experimental points to test ImmTAB-inhA efficacy against Mtb (H37Rv)-infected THP1 cells.

n°	Luminometry assays<br>Experimental points
1	Uninfected macrophages 24h
2	Infected macrophages 24h
3	Infected macrophages + PBMCs 24h
4	Infected macrophages + PBMCs + ImmTAB-inhA 1 nM 24h
5	Infected macrophages + PBMCs + ImmTAB-inhA 2.5 nM 24h
6	Infected macrophages + PBMCs + ImmTAB-inhA 5 nM 24h
7	Uninfected macrophages 48h
8	Infected macrophages 48h
9	Infected macrophages + PBMCs 48h
10	Infected macrophages + PBMCs + ImmTAB-inhA 1 nM 48h
11	Infected macrophages + PBMCs + ImmTAB-inhA 2.5 nM 48h
12	Infected macrophages + PBMCs + ImmTAB-inhA 5 nM 48h
13	Uninfected macrophages 72h
14	Infected macrophages 72h
15	Infected macrophages + PBMCs 72h
16	Infected macrophages + PBMCs + ImmTAB-inhA 1 nM 72h
17	Infected macrophages + PBMCs + ImmTAB-inhA 2.5 nM 72h
18	Infected macrophages + PBMCs + ImmTAB-inhA 5 nM 72h

SI References

1. Chiang TW, le Sage C, Larrieu D, Demir M, & Jackson SP (2016) CRISPR-Cas9(D10A) nickase-based genotypic and phenotypic screening to enhance genome editing. *Sci Rep* 6:24356.
2. Homolka S, Niemann S, Russell DG, & Rohde KH (2010) Functional genetic diversity among Mycobacterium tuberculosis complex clinical isolates: delineation of conserved core and lineage-specific transcriptomes during intracellular survival. *PLoS Pathog* 6(7):e1000988.
3. Walker A. and Treveil A., Find Peptide Orthologs. Github. https://github.com/Immunocore/FindPeptideOrthologs. Deposited 19 April 2024.
4. Liddy N, *et al.* (2012) Monoclonal TCR-redirected tumor cell killing. *Nat Med* 18(6):980-987.
5. Yang H, *et al.* (2016) Elimination of Latently HIV-infected Cells from Antiretroviral Therapy-suppressed Subjects by Engineered Immune-mobilizing T-cell Receptors. *Mol Ther* 24(11):1913-1925.
6. Boulter JM, *et al.* (2003) Stable, soluble T-cell receptor molecules for crystallization and therapeutics. *Protein Eng* 16(9):707-711.
7. Li Y, *et al.* (2005) Directed evolution of human T-cell receptors with picomolar affinities by phage display. *Nat Biotechnol* 23(3):349-354.
8. Winter G, *et al.* (2018) DIALS : implementation and evaluation of a new integration package. *Acta Crystallographica Section D Structural Biology* 74(2):85-97.
9. Beilsten-Edmands J, *et al.* (2020) Scaling diffraction data in the DIALS software package: algorithms and new approaches for multi-crystal scaling. *Acta Crystallogr D Struct Biol* 76(Pt 4):385-399.
10. Winter G (2009) xia2: an expert system for macromolecular crystallography data reduction. *Journal of Applied Crystallography* 43(1):186-190.
11. Walters LC, *et al.* (2018) Pathogen-derived HLA-E bound epitopes reveal broad primary anchor pocket tolerability and conformationally malleable peptide binding. *Nat Commun* 9(1):3137.
12. Le Nours J, *et al.* (2016) Atypical natural killer T-cell receptor recognition of CD1d-lipid antigens. *Nat Commun* 7:10570.
13. Liebschner D, *et al.* (2019) Macromolecular structure determination using X-rays, neutrons and electrons: recent developments in Phenix. *Acta Crystallogr D Struct Biol* 75(Pt 10):861-877.
14. Emsley P, Lohkamp B, Scott WG, & Cowtan K (2010) Features and development of Coot. *Acta Crystallogr D Biol Crystallogr* 66(Pt 4):486-501.
15. Murshudov GN, *et al.* (2011) REFMAC5 for the refinement of macromolecular crystal structures. *Acta Crystallogr D Biol Crystallogr* 67(Pt 4):355-367.
16. Winn MD, *et al.* (2011) Overview of the CCP4 suite and current developments. *Acta Crystallogr D Biol Crystallogr* 67(Pt 4):235-242.
17. Joosten RP, Joosten K, Murshudov GN, & Perrakis A (2012) PDB_REDO: constructive validation, more than just looking for errors. *Acta Crystallogr D Biol Crystallogr* 68(Pt 4):484-496.
18. Joosten RP, *et al.* (2009) PDB_REDO: automated re-refinement of X-ray structure models in the PDB. *J Appl Crystallogr* 42(Pt 3):376-384.

19. Bielecka MK, *et al.* (2017) A Bioengineered Three-Dimensional Cell Culture Platform Integrated with Microfluidics To Address Antimicrobial Resistance in Tuberculosis. *mBio* 8(1).
20. Joosten SA, *et al.* (2010) Mycobacterium tuberculosis peptides presented by HLA-E molecules are targets for human CD8 T-cells with cytotoxic as well as regulatory activity. *PLoS Pathog* 6(2):e1000782.
21. Pietra G, *et al.* (2003) HLA-E-restricted recognition of cytomegalovirus-derived peptides by human CD8+ cytolytic T lymphocytes. *Proc Natl Acad Sci U S A* 100(19):10896-10901.
22. Ulbrecht M, Modrow S, Srivastava R, Peterson PA, & Weiss EH (1998) Interaction of HLA-E with peptides and the peptide transporter in vitro: implications for its function in antigen presentation. *J Immunol* 160(9):4375-4385.
23. Molero-Abraham M, Lafuente EM, Flower DR, & Reche PA (2013) Selection of conserved epitopes from hepatitis C virus for pan-populational stimulation of T-cell responses. *Clin Dev Immunol* 2013:601943.
24. Michaelsson J, *et al.* (2002) A signal peptide derived from hsp60 binds HLA-E and interferes with CD94/NKG2A recognition. *J Exp Med* 196(11):1403-1414.
25. Wooden SL, Kalb SR, Cotter RJ, & Soloski MJ (2005) Cutting edge: HLA-E binds a peptide derived from the ATP-binding cassette transporter multidrug resistance-associated protein 7 and inhibits NK cell-mediated lysis. *J Immunol* 175(3):1383-1387.

Dataset S1 (separate file). Supporting dataset for mass spectrometry.

Sheet A – Cell line information including HLA genotypes for THP-1 and U937 cells.

Sheet B – Details on precursor on and fragment ions used in the quantitative assessment.

Sheet C – Extracted peak areas for all fragments and copy number calculations.

Dataset S2 (separate file). Contains the data of the donors and results from the different experimental conditions.

Movie S1 (separate file). Antigen-dependent killing of target cells in the presence of ImmTAB-inhA. Confocal imaging of PBMC (blue) co-cultured with a mixture of Ag+ (red; *inhA* transduced) and Ag– (yellow; non-transduced) A549 cells in the presence of 10 pM ImmTAB-inhA. Images were taken at 30-minute intervals over 3 days starting 7 h after addition of PBMC and displayed at 15 fps. Corresponding stills are shown in Figure 7B (top panels).

Movie S2 (separate file). No killing of target cells in the absence of ImmTAB-inhA. Confocal imaging of PBMC (blue) co-cultured with a mixture of Ag+ (red; inhA transduced) and Ag– (yellow; non-transduced) A549 cells in the absence of ImmTAB-inhA. Images were taken at 30-minute intervals over 3 days starting 7 h after addition of PBMC and displayed at 15 fps. Corresponding stills shown in Figure 7B (bottom panels).
